# BRD9 degraders as chemosensitizers in acute leukemia and multiple myeloma

**DOI:** 10.1038/s41408-022-00704-7

**Published:** 2022-07-19

**Authors:** Ellen Weisberg, Basudev Chowdhury, Chengcheng Meng, Abigail E. Case, Wei Ni, Swati Garg, Martin Sattler, Abdel Kareem Azab, Jennifer Sun, Barbara Muz, Dana Sanchez, Anthia Toure, Richard M. Stone, Ilene Galinsky, Eric Winer, Scott Gleim, Sofia Gkountela, Alexia Kedves, Edmund Harrington, Tinya Abrams, Thomas Zoller, Andrea Vaupel, Paul Manley, Michael Faller, BoYee Chung, Xin Chen, Philipp Busenhart, Christine Stephan, Keith Calkins, Debora Bonenfant, Claudio R. Thoma, William Forrester, James D. Griffin

**Affiliations:** 1 Department of Medical Oncology, Dana-Farber Cancer Institute, Boston, MA USA; 2 Department of Medicine, Harvard Medical School, Boston, MA USA; 3 Washington University in Saint Louis School of Medicine, St. Louis, MO USA; 4 Novartis Pharma AG, Basel, Switzerland; 5 Alphina Therapeutics, Westport, CT USA

**Keywords:** Targeted therapies, Cancer

## Abstract

Bromodomain-containing protein 9 (BRD9), an essential component of the SWI/SNF chromatin remodeling complex termed ncBAF, has been established as a therapeutic target in a subset of sarcomas and leukemias. Here, we used novel small molecule inhibitors and degraders along with RNA interference to assess the dependency on BRD9 in the context of diverse hematological malignancies, including acute myeloid leukemia (AML), acute lymphoblastic leukemia (ALL), and multiple myeloma (MM) model systems. Following depletion of BRD9 protein, AML cells undergo terminal differentiation, whereas apoptosis was more prominent in ALL and MM. RNA-seq analysis of acute leukemia and MM cells revealed both unique and common signaling pathways affected by BRD9 degradation, with common pathways including those associated with regulation of inflammation, cell adhesion, DNA repair and cell cycle progression. Degradation of BRD9 potentiated the effects of several chemotherapeutic agents and targeted therapies against AML, ALL, and MM. Our findings support further development of therapeutic targeting of BRD9, alone or combined with other agents, as a novel strategy for acute leukemias and MM.

## Introduction

Acute myeloid leukemia (AML), characterized by clonal expansion of undifferentiated myeloid progenitor cells, is the most common acute leukemia affecting adults, with an incidence of over 20,000 cases annually in the U.S. [1–3]. The prognosis for AML varies depending on mutational drivers, karyotype complexity and age of patient, with an overall survival (OS) rate at 5 years for patients <55 years ranging from “favorable” (OS rate = 64%), to “intermediate” (OS rate = 41%), to “adverse” (OS rate = 11%) [1]. The incidence of AML rises with age, and older patients (>65 years), who comprise most newly diagnosed cases, often cannot tolerate available therapies and have a particularly dismal prognosis [3]. Thus, AML is a complex disease and remains an unmet medical need. Recent drug approvals for patients harboring gain-of-function mutations in FLT3 and IDH1/2 include midostaurin, gilteritinib, ivosidenib & enasidenib, and suggest that fundamental understanding of disease subtypes will drive the development of targeted and more effective medicines.

New therapeutic approaches are also needed for non-AML hematopoietic malignancies, including older patients afflicted with acute lymphoblastic leukemia (ALL), and multiple myeloma (MM). The majority of ALL cases, characterized by proliferation of malignant, immature lymphatic blasts [4], occur in children and are mostly curable; however, the prognosis for adults is poor due at least in part to chemotherapy resistance and drug intolerance [5]. MM, a cancer of clonal plasma cells, is the second most common hematologic malignancy after lymphoma [6, 7]. Standard treatments, including proteasome inhibitors and immunomodulatory drugs (IMiDs), have improved overall 5-year survival rates to 49–56% and still provide an opportunity for agents that could provide durable remissions and cures [7].

Epigenetic dysregulation is directly associated with multiple hematological cancers [8]. Mutations in DNMT3A, NPM1, CBFβ/RUNX, splicing factors and the chromosome cohesion machinery (STAG2, RAD21, SMC1A/3) [9] provide insight that myeloid differentiation requires remodeling, at the level of chromosomes, chromatin and/or gene control. Components of SWI/SNF chromatin remodeling complexes have also been identified as dependencies in AML, which may constitute a new treatment approach. In particular, the bromodomain-containing protein 9 (BRD9), a specific component of the non-canonical (nc-BAF/GBAF) mammalian SWI/SNF chromatin remodeling complex [10–12], is essential to maintain the transformed phenotype of AML. AML cells sensitive to loss of BRD9 undergo cell cycle arrest and differentiate. While distinct SWI/SNF (BAF) complexes have been described that additionally include canonical BAF (cBAF) and polybromoBAF (PBAF), there appears to be a specific dependency on BRD9 for the completion of myeloid differentiation in a subset of AML cell lines. Therefore while the BAF complexes regulate key steps in chromatin transactions required for cell fate decisions, including transcription factor binding and gene expression, the BRD9-containing BAF complex appears to play key roles in myeloid leukemia. [10, 13–15].

Previous work identified nc-BAF subunits as synthetic lethal targets in malignant rhabdoid tumors and synovial sarcoma that harbor driver mutations in core components of SWI/SNF complexes; these findings suggest that an essential role for BRD9 reflects an interplay among these complexes arising from these mutations [16]. As noted, BRD9 has also been found to be important for AML cell proliferation [17, 18], despite the absence of reported mutations in SWI/SNF subunits. This may reflect a stage-specific dependence of BRD9 to maintain a myeloid progenitor program, perhaps at the level of maintaining expression of a progenitor or oncogenic program as described [17–19].

A number of small-molecule inhibitors of BRD9, based on a phenyl naphthyridone scaffold (BI7273 & BI9564), have been found to inhibit proliferation of mouse and human AML cell lines [17, 20]. Paradoxically, the bromodomain in BRD9 has been shown to be an ineffectual drug target and degradation is required to reproduce the dependency seen with shRNA and CRISPR screens [21]. In this report, we describe a novel BRD9 bromodomain inhibitor, EA-89-YM35 (or EA-89), and a degrader-based analog (QA-68-ZU81, or QA-68) that incorporates the EA-89 warhead into a cereblon (CRBN)-targeting proteolysis-targeting chimera (PROTAC), to compare the efficacy of inhibition versus degradation to the impact seen with BRD9 knock-down (KD) by RNA interference. We also explore the potential for BRD9 targeting to enhance antileukemic effects of chemotherapeutic agents and targeted inhibitors as a novel therapeutic strategy for acute leukemia and MM. Our findings indicate that the degradation of BRD9 results in significantly greater effects compared to bromodomain inhibitors, as seen in AML as well in other hematologic malignancies.

## Materials and Methods

All materials and methods details are provided in the Supplementary Data Section.

## Results

### BRD9 degrader treatment leads to inhibition of AML cell line proliferation

**Fig. 1:**
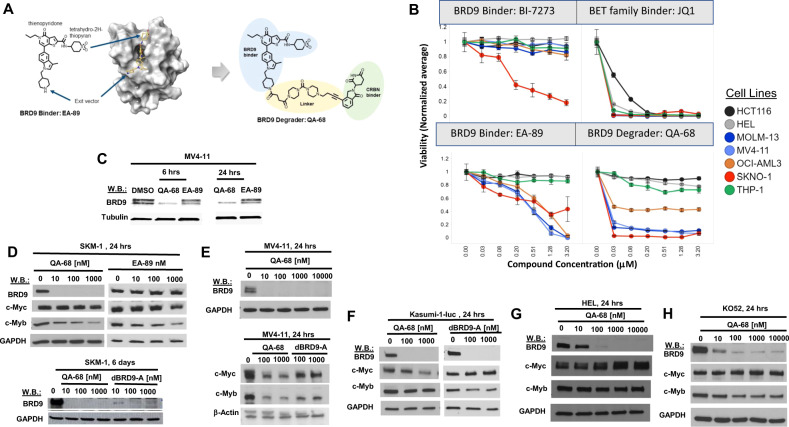
The BRD9 degrader, QA-68-ZU81, shows increased potency and selectivity versus BRD9 inhibitors. **A** Structure-based drug design evolves chemical matter from selective binding domain (BD) inhibitor, EA-89-YM35 (“EA-89”), to selective BD degrader, QA-68-ZU81 (“QA-68”). X-ray co-crystal shows binding mode of EA-89 on BRD9 at 1.76 Angstroms making extended interactions across the surface of BRD9 with thienopyridone docking into the acetyl lysine pocket. **B** The BRD9 degrader, QA-68, provides an >100-fold increase in potency versus the matched inhibitor, EA-89. 7-day assay. **C** Effects of BRD9 degrader treatment on BRD9 expression in MV4-11 cells. **D**, upper panel Comparison of effects of QA-68 and EA-89 on BRD9, c-MYC and c-MYB expression in SKM-1 cells. 24-h assay. **D**, lower panel Comparison of effects of QA-68 and dBRD9-A on BRD9 expression. 6-day assay. **E**–**H** Effects of QA-68 or dBRD9-A treatment on BRD9, c-Myc and c-Myb expression in MV4-11 (**E**), Kasumi-1-luc + (**F**), HEL (**G**) and K052 (**H**). 24-h assay.

BRD9 has been identified as a target in human AML using genetic screens [17]. To explore this further, we investigated the antileukemic activity of a novel small molecule inhibitor of BRD9 as well as PROTACs, in which this warhead is incorporated into a heterobifunctional degrader that directs ubiquitination and degradation of BRD9. EA-89 is a potent and selective inhibitor that binds to BRD9 in a novel way. The binding mode of EA-89 determined by X-ray crystallography reveals that the thienopyridone head group binds to the acetyl lysine pocket similar to other known bromodomain chemotypes, forming a direct hydrogen bond with the conserved Asn100. Propyl substitution off the pyridone nitrogen accesses an adjacent induced hydrophobic pocket while the tetrahydro-2H-thiopyran ring picks up additional protein interactions, not observed with previous BI-based PROTACs, on the opposite side of the acetyl lysine pocket (Fig. 1A). QA-68, an IMiD-based derivative of EA-89, extends from the piperidine nitrogen and includes a di-piperizine amide linker with an alkyne bridge to an IMiD that serves as a CRBN anchor (chemical synthesis details and information regarding EA-89 binding to BRD9 are provided in supplementary materials and methods).

We compared the activity of these molecules to a previously described potent and selective BRD9 inhibitor (non-degrader), BI-7273 [20], against human leukemia cell lines MV4;11 (B-myelomonocytic leukemia), EOL1 (eosinophilic leukemia), MOLM13 (acute monocytic leukemia), OCI-AML3 (AML), HEL (erythroleukemia), THP1 (acute monocytic leukemia) and SKNO1 (AML) (Fig. 1B). HCT116 (colorectal cancer) was included as an out-of-lineage control, and JQ1, an established potent inhibitor of BET family proteins BRD2, 3, 4, was included as an inhibitor of bromodomain proteins with a distinct selectivity profile. The inhibitor, EA-89, (structure shown in Fig. 1A) produces a strongly selective profile across a subset of leukemia lines with no impact on the viability of THP-1, HEL, or HCT116 (Fig. 1B). Interestingly, the degrader, QA-68, provides an >100-fold increase in potency over EA-89 (Fig. 1B) and robustly degrades BRD9 in AML cells (Fig. 1C–H). Of note, in addition to showing less activity toward HCT-116 cells, QA-68 also shows less antileukemic activity against the AML cell lines, HEL and THP-1, which suggests that QA-68 is not perfectly selective for AML and shows differential potency. The previously described inhibitor, BI-7273 [20], appears to be inactive across all lines with the exception of SKNO1, whereas JQ1 is potently antiproliferative across all lines, including the non-hematopoietic HCT116 (Fig. 1B).

**Fig. 2:**
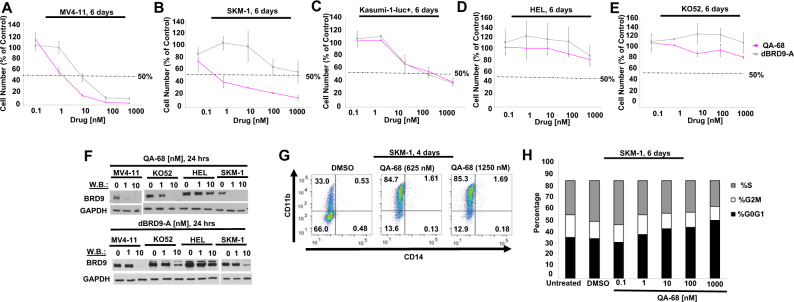
BRD9 degrader treatment leads to inhibition of AML cell line proliferation. **A**–**E** Proliferation assays: AML cell lines treated with QA-68 or dBRD9-A. 6-day assay. Proliferation studies shown are representative of independent studies performed at least twice for which similar results were observed. **F** Effects of QA-68 (upper panel) and dBRD9-A (lower panel) on BRD9 expression in QA-68-sensitive and –insensitive AML cell lines. 24-hour assay. **G** Effects of QA-68 on SKM-1 cell differentiation. 4-day assay. **H** Effects of QA-68 on SKM-1 cell cycle progression. 6-day assay.

dBRD9-A is a previously-described BRD9 degrader with activity in leukemia [22]. We compared its activity to these novel BRD9 ligands against a panel of leukemia lines. QA-68 treatment led to a concentration-dependent inhibition of proliferation of MV4-11, SKM-1 and Kasumi-1-luc+ cells (IC50 = 1–10 nM for MV4;11 and SKM-1, and IC50 = 10–100 nM for Kasumi-1-luc + ), whereas cell lines, such as HEL, KO52 and THP-1, showed less sensitivity at up to 6 days of treatment (Fig. 2A–E, Supplementary Fig. 1A–J). AML cell lines were generally less sensitive to EA-89, however, exhibited differential sensitivity to dBRD9-A (Fig. 2A–E, Supplementary Fig. 1A–E). A positive correlation was generally observed between the extent to which BRD9 was degraded and the sensitivity of AML cells to degrader treatment (Fig. 2F). However, this was not observed in the KO52 cell line, as BRD9 was absent at 10 nM QA-68, although there was no decrease in cell number at that dose (Fig. 2E). BRD9 degradation was observed in as little as 6 hours (Fig. 1C) and persists through 24 hours (Fig. 1C–H) and was sustained over 6 days of treatment (Fig. 1D, lower panel).

Based on previous reports [17], we investigated the effect of targeted BRD9 degradation in AML on the transcription factors c-MYC and c-MYB, both involved in myeloid cell growth, differentiation and survival. Concomitant with BRD9 degradation we observed decreases in c-MYC and c-MYB protein in MV4-11 cells, decreases in c-MYC only in Kasumi-luc+ cells, and decreases in c-MYB only in SKM-1 cells (Fig. 1D–F). In contrast, there were little to no changes in c-MYC or c-MYB expression in BRD9 degrader-treated HEL or KO52 cells (Fig. 1G, H).

### Targeted loss of BRD9 protein in AML cells affects cell growth, differentiation, and colony formation

BRD9 has previously been reported to maintain leukemia cells in a proliferating and undifferentiated state [17]. We observed BRD9 degraders to induce differentiation of SKM-1 cells, in contrast to the BRD9 inhibitor, EA-89, which had little effect on SKM-1 differentiation and was less potent in inhibiting cell proliferation (Fig. 2G, Supplementary Fig. 2A, B, Supplementary Fig. 3A, B). In addition, QA-68 (degrader) more effectively inhibited SKM-1 cell cycle progression than EA-89 (Fig. 2H and Supplementary Fig. 2C). Concentrations of QA-68 used in the differentiation and cell cycle studies were sufficient to degrade BRD9, and concentrations of EA-89 used for these studies were sufficient to decrease levels of c-MYB, suggesting an active and on-target concentration range for both drugs (Fig. 1D).

**Fig. 3:**
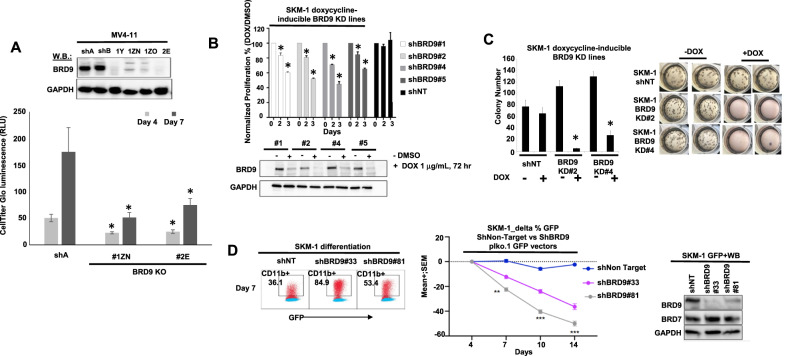
Targeted loss of BRD9 protein in AML cells due to genetic ablation affects cell growth, differentiation, and colony formation. **A** KO of BRD9 in MV4-11 cells correlates with inhibited cell proliferation. Asterisks represent statistical significance. t-test: ShA:BRD9 KO clones (*P* < 0.0001). **B** Inducible KD of BRD9 in SKM-1 cells correlates with inhibited cell proliferation. Asterisks represent statistical significance. t-test: shNT:shBRD9 KD clones (*P* < 0.0001). **C** Effects of doxycycline-inducible BRD9 KD on SKM-1 colony formation. 200 cells/mL/well were seeded using MethoCult Classic (+cytokines) and colonies were counted 7 days postseeding. Colonies were counted in 2–3 wells and averages + /- standard deviations were determined. (Left panel) Colony counts shown as bar graphs. Asterisks represent statistical significance. t-test: SKM-1 control (-dox): SKM-1 control (+dox) (*P* = 0.237); SKM-1 BRD9 KD#2 (-dox):SKM-1 BRD9 KD#2 (+dox) (*P* = 0.0001); SKM-1 BRD9 KD#4 (-dox):SKM-1 BRD9 KD#4 (+dox) (*P* = 0.0001). (Right panel) Images of colonies. **D** Effects of BRD9 KD on SKM-1 cell differentiation (from a pool of triplicates) (left panel), and growth (6 replicates for each time point) (middle panel). Y-axis shows percent GFP positive cells. (Right panel) BRD9 and BRD7 expression in BRD9 KD cells versus nontarget KD control cells.

To validate BRD9 as a target important for viability and differentiation of AML cells, we performed Crispr knockout (KO) and doxycycline-induced knockdown (KD) of BRD9 in selected AML cell lines. We observed a correlation between Crispr KO of BRD9 or doxycycline-induced BRD9 KD and inhibition of AML cell growth (Fig. 3A, B). In addition, doxycycline-induced BRD9 KD led to inhibition of AML cell colony growth in a methylcellulose assay (Fig. 3C). An alternative shRNA vector system using plko.1 GFP led to induction of SKM-1 differentiation following KD of BRD9, as measured by CD11b expression, and inhibition of SKM-1 cell growth (Fig. 3D), as well as inhibition of MV4-11 cell proliferation (Supplementary Fig. 4A, B). Importantly, BRD9 was selectively targeted without any loss of BRD7, suggesting that effects of KD on growth and differentiation were on-target and due to BRD9 depletion. Taken together, our results confirm a BRD9 dependency in AML.

### Targeted loss of BRD9 protein affects ALL cell growth and viability

**Fig. 4:**
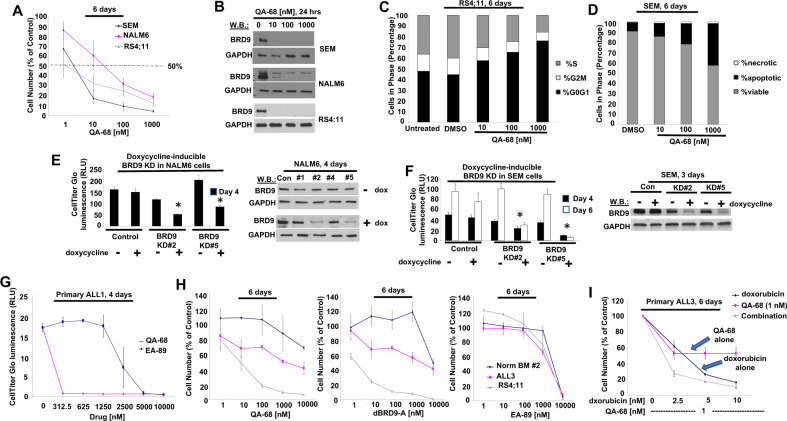
Targeted loss of BRD9 protein inhibits ALL cell proliferation and cell cycle progression and decreases viability. **A** Proliferation assays: ALL cell lines treated with QA-68. 6-day assay. **B** Effects of QA-68 on BRD9 expression in ALL cell lines. 24-h assay. **C** Effects of QA-68 on RS4;11 cell cycle progression. 6-day assay. **D** Effects of QA-68 SEM cell apoptosis. 6-day assay. **E**, left panel Proliferation of NALM6 control cells and doxycycline-inducible BRD9 KD NALM6 cells + /-doxycycline. 4-day assay. Asterisks indicate statistical significance. t-test, 4-day: NALM6 control (-dox):NALM6 control (+dox) (*P* = 0.377); NALM6 BRD9 KD#2 (-dox):NALM6 BRD9 KD#2 (+dox) (*P* < 0.0001); NALM6 BRD9 KD#5 (-dox):NALM6 BRD9 KD#5 (+dox) (*P* = 0.0212). (**E**, right panel) BRD9 expression in NALM6 control cells and doxycycline-inducible BRD9 KD NALM6 cells + /-doxycycline. **F**, left panel Proliferation of SEM control cells and doxycycline-inducible BRD9 KD SEM cells + /-doxycycline. 4- and 6-day assays. t-test, 4-day: SEM control (-dox):SEM control (+dox) (*P* = 0.2879); SEM BRD9 KD#2 (-dox):SEM BRD9 KD#2 (+dox) (*P* = 0.0107); SEM BRD9 KD#5 (-dox):SEM BRD9 KD#5 (+dox) (*P* = 0.0004). t-test, 6-day: SEM control (-dox):SEM control (+dox) (*P* = 0.1924); SEM BRD9 KD#2 (-dox):SEM BRD9 KD#2 (+dox) (*P* = 0.0006); SEM BRD9 KD#5 (-dox):SEM BRD9 KD#5 (+dox) (*P* = 0.0003). (**F**, right panel) BRD9 expression in SEM control cells and doxycycline-inducible BRD9 KD SEM cells + /-doxycycline. DOX, 1 µg/ml; DMSO, 0.1%. **G** Proliferation assays: Primary ALL1 cells treated with QA-68 and EA-89 for 4 days. **H** Proliferation assays: Normal bone marrow cells (sample #2) and primary ALL3 cells treated with QA-68 (left panel), dBRD9-A (middle panel), or EA-89 (right panel). 6-day assay. RS4;11 cells were tested as a positive control. **I** Treatment of primary ALL-3 cells with QA-68 alone, doxorubicin alone, or a combination. 6-day assay.

Given the observed importance of BRD9 for the viability and growth of AML cells, we hypothesized that a BRD9 dependency might similarly exist for other hematopoietic malignancies. To address this, we tested the activity of BRD9 degraders, QA-68 and dBRD9-A, on the growth and viability of a panel of human ALL cell lines. All B-ALL cell lines that we tested showed different levels of sensitivity to BRD9 degrader treatment: SEM, SEMK2, RS4;11, and REH (IC50s in the range of 1–10 nM); 697 and p190BCR-ABL-positive SUP-B15 (IC50s close to 1 nM); NALM6 and RCH-ACV (IC50s in the 10–100 nM range) (Fig. 4A, Supplementary Figs. 5A–E and 6A–D). Drug treatment-induced BRD9 degradation correlated with the drug sensitivity of the ALL cell lines: for example, partial BRD9 degradation was observed in less BRD9 degrader-sensitive NALM6 cells whereas BRD9 degradation was complete for more BRD9 degrader-sensitive lines such as RS4;11 and SEM (Fig. 4A, B, Supplementary Fig. 5A–E). As was observed with AML, the BRD9 degraders, QA-68 and dBRD9-A, showed higher potency against the B- ALL lines than the BRD9 inhibitor, EA-89 (Supplementary Fig. 5E, F, Supplementary Fig. 6A–D). BRD9 degrader effects on c-MYC expression, similar to AML, were variable and modest among the cell lines tested (Supplementary Fig. 5). In contrast to the high BRD9 degrader sensitivity we observed for B-ALL lines, human T-ALL cell lines, including PF-382, Jurkat, CCRF-CEM, DND-41, HPB-ALL, KOPT-K1, and MOLT-4, were insensitive in terms of growth inhibition (Supplementary Fig. 6E–L). BRD9 degrader treatment led to G1 arrest in some B-ALL cell lines, and induction of apoptosis in other cell lines (Fig. 4C, D and data not shown). Our results collectively suggest that B-ALL is a hematopoietic malignancy that is sensitive to BRD9 degrader treatment and leads to a cytotoxic or cytostatic response.

To validate BRD9 as a target important for proliferation and viability of ALL cells, we performed doxycycline-induced KD of BRD9 in selected ALL cell lines. We observed a correlation between doxycycline-induced BRD9 KD and inhibition of NALM6 and SEM cell line growth (Fig. 4E, F). These results are supportive of a BRD9 dependency in ALL and introduce ALL as a novel disease target for BRD9 degrader therapy.

To investigate the clinical potential of BRD9 targeting through degradation or inhibition for ALL, we tested BRD9 degraders and the BRD9 inhibitor, EA-89, against primary ALL cells (patient details are provided in Supplementary Table 1). Primary ALL cells showed more sensitivity to QA-68 and dBRD9-A than normal bone marrow cells, and more potency than EA-89 against primary ALL cells (primary ALL1 and primary ALL3) (Fig. 4G, H). In addition, QA-68 potentiated the effects of daunorubicin against primary ALL cells and RS4;11 cells (tested as a control) (Fig. 4I and Supplementary Fig. 7A, B).

### Targeted loss of BRD9 protein inhibits primary AML cell growth and colony formation

**Fig. 5:**
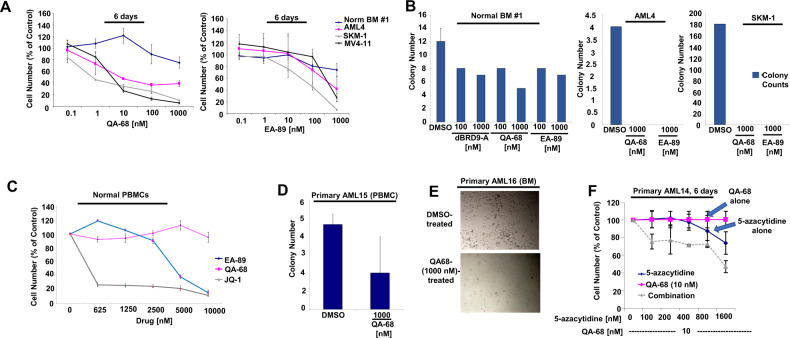
Targeted loss of BRD9 protein by degradation leads to inhibition of primary AML and ALL cell growth and colony formation. **A** Proliferation assays: Normal bone marrow cells (sample #1) and primary AML4 cells (secondary AML) treated with QA-68 and EA-89. 6-day assay. SKM-1 and MV4-11 cell lines were tested as positive controls (**B**) QA-68, EA-89 and dBRD9-A effects on normal bone marrow or primary AML4 colony formation using MethoCult H4434 Classic methylcellulose medium (with cytokines) and IMDM culture media. SKM-1 cells were tested as a positive control. Shown are colony counts taken following 8 days from the start of the assay. **C** Treatment of normal PBMCs with QA-68 or EA-89 versus the BRD4 inhibitor, JQ1. 4-day assay. **D** Effects of QA-68 treatment on primary AML PBMCs (sample AML15) colony formation, using MethoCult Enriched H4435 methylcellulose medium and IMDM culture media. Shown are colony counts taken following 12 days from the start of the assay. **E** Effects of QA-68 treatment on primary AML patient BM cells (sample AML16) colony formation, using MethoCult Enriched H4435 methylcellulose medium and IMDM culture media supplemented with cytokine cocktail and FLT3L. Shown are representative images from a well following 6 days from the start of the assay. **F** Treatment of primary AML cells (sample AML14) with QA-68 alone, 5-azacytidine alone, or a combination. 6-day assay.

To assess the clinical potential of BRD9 targeting for AML, we investigated the antileukemic activity of QA-68 and EA-89 against primary AML cells (patient details provided in Supplementary Table 1), using proliferation and colony formation assays (Fig. 5). Primary AML cells (AML4), SKM-1 and MV4-11 cells, showed higher sensitivity to QA-68 treatment than normal bone marrow cells and thus, importantly, demonstrated a therapeutic window for QA-68 (Fig. 5A and Supplementary Fig. 7C). Consistent with AML cell lines, QA-68 was more potent against primary sample AML4 than EA-89 (Fig. 5A). When tested in colony formation assays, BRD9 degrader- or inhibitor-treated primary AML cells completely failed to grow whereas colony formation with normal bone marrow-derived cultures were decreased by a modest 2-fold effect (Fig. 5B). In addition, treatment of normal peripheral blood mononucleated cells (PBMCs) with QA-68 or the BRD2,3,4 inhibitor, JQ1, at concentrations up to 10 μΜ showed no detectable cell loss by QA-68, while JQ1 produced a strong viability phenotype (Fig. 5C). EA-89, while less toxic toward normal PBMCs than JQ1, did however kill cells at higher concentrations (5–10 μM). Coupled with the lack of toxicity of BRD9 degrader treatment against normal bone marrow cells, our findings suggest small molecule degradation of BRD9 is comparatively nontoxic toward normal hematopoietic cells. Of note, QA-68 treatment leads to degradation of BRD9, but not the JQ1 target, BRD4, in leukemia cells, and washout of QA-68 leads to a reversal of BRD9 degradation effects but has no effect on BRD4 (Supplementary Fig. 8A, B). Taken together, these results support the selectivity of QA-68 toward BRD9 as a protein target.

QA-68 was observed to inhibit colony formation of additional primary AML bone marrow and PBMC cells (Fig. 5D, E). Interestingly, despite QA-68 and 5-azacytidine showing little potency against one primary AML sample (AML14) as single agents, the combination of QA-68 and 5-azacytidine was more efficacious, resulting in a leftward shift of the dose-response curves (Fig. 5F).

### Targeted loss of BRD9 protein in MM inhibits cell growth, viability, cell cycle progression, and colony formation

**Fig. 6:**
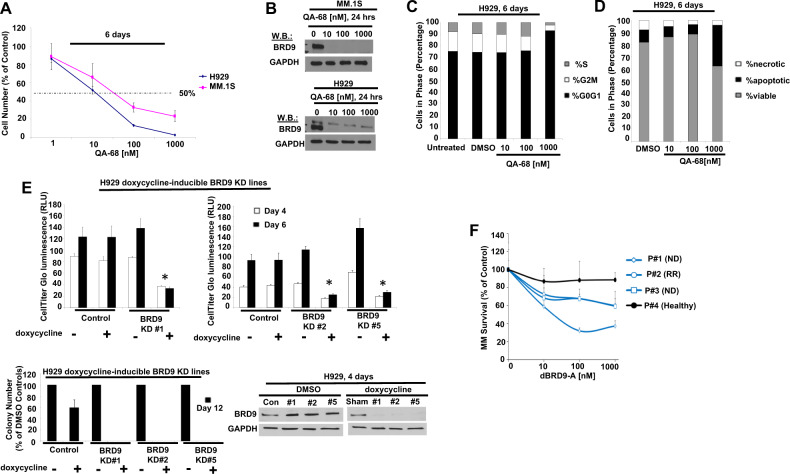
Targeted loss of BRD9 protein in MM leads to inhibition of cell proliferation, cell cycle progression, and colony formation and a decrease in viability. **A** Proliferation assays: MM.1S or H929 cells treated with QA-68. 6-day assay. **B** Effects of QA-68 on BRD9 expression in MM.1S and H929 cells. 24-hour assay. **C** Effects of QA-68 on H929 cell cycle progression. 6-day assay. **D** Effects of QA-68 on H929 apoptosis. 6-day assay. **E**, upper panels Proliferation of H929 control cells and doxycycline-inducible BRD9 KD H929 cells + /-doxycycline. 4- and 6-day assays. Asterisks indicate statistical significance. **E**, upper left panel: t-test, 4-day: H929 control (-dox): H929 control (+dox) (*P* = 0.1761); H929 BRD9 KD#1 (-dox): H929 BRD9 KD#1 (+dox) (*P* < 0.0001). **E**, upper right panel: t-test, 4-day: H929 control (-dox):H929 control (+dox) (*P* = 0.2475); H929 BRD9 KD#2 (-dox):H929 BRD9 KD#2 (+dox) (*P* < 0.0001); H929 BRD9 KD#5 (-dox):H929 BRD9 KD#5 (+dox) (*P* < 0.0001). **E**, upper left panel: t-test, 6-day: H929 control (-dox):H929 control (+dox) (*P* = 0.9899); H929 BRD9 KD#1 (-dox):H929 BRD9 KD#1 (+dox) (*P* = 0.0004). **E**, upper right panel: t-test, 6-day: H929 control (-dox):H929 control (+dox) (*P* = 0.9316); H929 BRD9 KD#2 (-dox):H929 BRD9 KD#2 (+dox) (*P* < 0.0001); H929 BRD9 KD#5 (-dox): H929 BRD9 KD#5 (+dox) (*P* < 0.0003). **E**, lower left panel Effects of doxycycline-inducible BRD9 KD on H929 colony formation. 1000 cells/mL/well were seeded; colonies were counted 12 days postseeding. Colonies were counted twice/well. Data are presented as percent of DMSO-treated (-doxycycline) control wells. **E**, lower right panel BRD9 expression in H929 control cells and doxycycline-inducible BRD9 KD H929 cells + /- doxycycline. DOX, 1 µg/ml; DMSO, 0.1%. **F** Effects of BRD9 degrader treatment on proliferation of three primary MM patient samples versus one normal/healthy bone marrow sample, cultured in 3D tissue engineered bone marrow (3DTEBM). 5-day assay. MM bone marrow aspirates were treated with dBRD9-A (concentration range 10–1000 nM; compounds added inside 3D and outside 3D); MM survival was tested by flow cytometry (cell number normalized to counting beads, and then normalized to control). ND Newly Diagnosed, RR Relapsed/Refractory.

We were interested in investigating other hematopoietic malignancies to determine what role, if any, BRD9 may play in growth and viability. We treated several MM cell lines with QA-68, dBRD9-A, and EA-89 and, similar to AML and ALL, we observed QA-68 or dBRD9A to more potently inhibit cell proliferation than EA-89 with a concomitant decrease in BRD9 protein (Fig. 6A, B and Supplementary Fig. 9). Cell lines, including MM.1S and H929, showed the highest sensitivity to BRD9 degrader treatment as compared to other lines, such as U266 and 8226. QA-68- and dBRD9-A-induced cell killing correlated with BRD9 degradation, and as observed with AML and ALL, c-MYC expression was variable in response to BRD9 loss in different MM lines (Supplementary Fig. 9D, F). QA-68 or dBRD9-A treatment of MM cell lines induced modest levels of apoptosis and inhibited cell cycle progression (most apparent at 1000 nM) (Fig. 6C, D). Doxycycline-induced BRD9 KD in H929 cells led to inhibited cell proliferation and inhibition of colony formation, validating BRD9 as an important target for the growth and viability of MM cells (Fig. 6E). Importantly, dBRD9-A selectively killed primary MM cells, with little effect on bone marrow cells from a healthy donor (patient details provided in Supplementary Table 1) (Fig. 6F).

### Targeted inhibition or degradation of BRD9 potentiates the antileukemic effects of standard chemotherapy and targeted agents against acute leukemia and MM

**Fig. 7:**
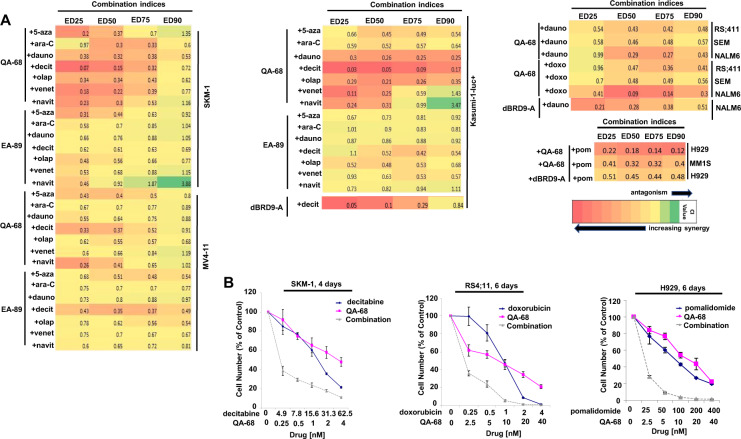
Targeted inhibition or degradation of BRD9 potentiates the antiproliferative effects of chemotherapy agents and targeted therapies on AML, ALL and MM. **A** Combination indices generated from combination studies investigating the effects of QA-68 or EA-89 alone or combined with chemotherapy or targeted agents against AML cell lines (left and middle panels) and ALL and MM cell lines (right panel). **B** Representative dose-response curves showing antiproliferative effects of chemotherapy alone, QA-68 alone, or a combination against SKM-1, RS4;11, and H929 cells. Proliferation studies shown are representative of independent studies performed at least twice for which similar results were observed.

The heterogeneity of AML, attributed to multiple factors, can enable the establishment of leukemic clones with distinct dependencies at different stages of myeloid differentiation making AML a therapeutically challenging disease. Combination therapy is an approach used to increase remissions across diverse AML subtypes. We therefore investigated the ability of BRD9 targeting to potentiate agents that are the standard of care in leukemia, such as decitabine, ATRA, and 5-azacytidine, as well as more targeted agents, such as venetoclax and olaparib. We observed QA-68, dBRD9-A, and EA-89 to display high synergizing activity as measured by antiproliferative activity with the observation that both degraders when combined with other agents induce differentiation (Fig. 7A, B, Supplementary Fig. 10, and Supplementary Fig. 11). The degree of synergy observed with QA-68, as assessed by combination indices generated using Chou and Talalay analysis [23], was stronger than that observed with EA89 (Fig. 7A, B).

We next investigated the ability of BRD9 degrader treatment to potentiate effects of standard of care therapy against ALL and MM. We observed QA-68 or dBRD9-A to synergize with standard ALL therapies, such as the anthracyclines daunorubicin and doxorubicin, against several B-ALL lines, including RS4;11, SEM, and NALM6, as well as to synergize with pomalidomide against MM cells (Fig. 7A, B). Together, our results support BRD9 targeting as a potential therapeutic strategy for ALL and MM in combination with standard of care agents.

### BRD9 regulates common and lineage-dependent pathways in heme malignancies, including those associated with cell cycle progression, the inflammatory response, immune response, cell adhesion, and DNA replication and repair

**Fig. 8:**
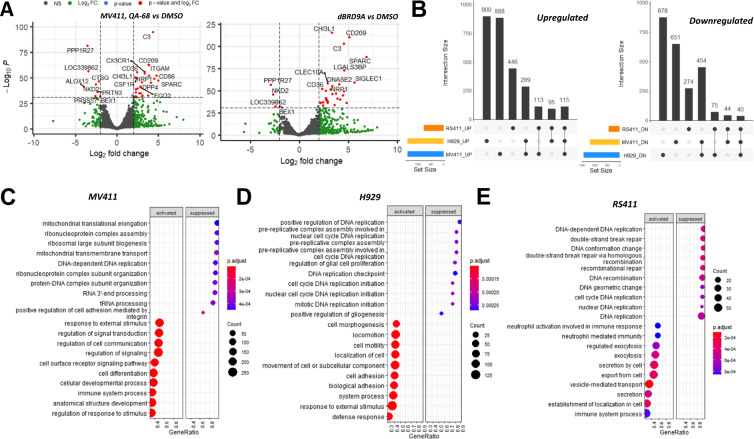
Targeted loss of BRD9 leads to deregulation of major signaling pathways in hematopoietic malignancies, including cell cycle progression, the inflammatory response, immune response, cell adhesion and DNA replication and repair. **A** Global transcript perturbation profile after QA-68 or dBRD9-A treatment in MV411 was contrasted. Genes undergoing |log2FC | > 2 and p < 10e-32 displayed as red dots. **B** Intersection sets of the number of differentially expressed genes (DEGs) that are unique and conserved after QA-68 treatment in MV4-11, H929 and RS4;11 cells. (**C**–**E**) Gene Set Enrichment Analysis of QA-68 treatment induced DEGs in MV4-11 (**C**), H929 (**D**), and RS4;11 (**E**) cells. DEGs are defined by Benjamini & Hochberg FDR < 0.05 & |FC | > 1.5 between QA-68 and DMSO treatments in respective cell lineages.

Using RNA-seq analysis, the global transcript perturbation profile after QA-68 or dBRD9-A treatment in MV4-11 cells, as a representative of AML, was compared. Principal component analysis of the dBRD9-A-treated group and the novel QA-68-treated group accounts for over 80% of the variance when compared to the DMSO treatment, while the inter-degrader group variance <5% (Fig. 8A and Supplementary Fig. 12A). In addition to AML, we compared the effects of QA-68 treatment (24 hr, 1 μM, which is nontoxic to normal bone marrow cells) on gene transcription in RS411 and H929 as representative of ALL and MM lineages. We observed more pronounced perturbation (Fig. 8B) in gene expression by QA-68 treatment in MV4-11 and H929 than in RS4;11 (Fold change 1.5 and Benjamini-Hochberg False discovery rate < 0.05). Geneset enrichment test in MV4-11 and H929 was in agreement with previously published reports that loss of BRD9 supported repression of the MYC pathway [17], while induction of myeloid differentiation signatures like ITGAM, PRDM1, TLR5, MAFB, FOS, CSF1R and ID2 (Fig. 8A–D and Supplementary Fig. 12B) was unique to MV4-11. In all three hematopoietic malignancies, we observe upregulation of Differentially Expressed Genes (DEGs) enriched in biological processes associated with signal transduction, inflammation, immune response, and cell adhesion (Fig. 8C–E and Supplementary Fig. 12B). Downregulated pathways in common among the three malignancies displayed that BRD9 targeting perturbed the underlaying signatures critical to cell survival in these diseases and included cell cycle progression, and DNA repair and replication. Not unexpectedly, the largest number of DEGs were non-overlapping and unique to each of the three hematopoietic malignancies investigated (Fig. 8B). The analysis revealed 40 genes to be commonly downregulated in AML, ALL and MM, and 115 genes to be commonly upregulated in the three malignancies (Fig. 8B). qPCR was employed to validate the deregulation of selected genes in response to BRD9 degrader treatment (Supplementary Fig. 12C–E).

## Discussion

BRD9, a subunit of the ncBAF SWI/SNF chromatin remodeling complex, has previously been reported to be important for the growth and viability of AML cells [17, 20], and BRD9-targeting degraders have been developed and evaluated as a novel therapeutic strategy for AML [21, 22, 24]. The main theme of the current study is BRD9 as a therapeutic, druggable target in a select panel of heme malignancies that includes and extends beyond AML, previously identified as exhibiting BRD9 dependency and thus serving as a positive reference for comparison with the other blood cancers we investigated. Specifically, we examine the importance of BRD9 for AML cell survival benchmarking a novel BRD9 degrader against a previously reported BRD9 degrader as well as BRD9 suppression by KO and KD approaches. Our findings reveal a clear BRD9 dependency in AML as well as in two additional hematologic malignancies, B-ALL and MM. Interestingly we also observe a consistent lack of response in malignancies of the T lymphocytic lineage.

Our results show a similar pattern among all three heme malignancies. For AML, we observe a correlation among several lines between sensitivity to BRD9 degrader treatment in terms of inhibition of cell growth and the extent of BRD9 degradation (MV4-11, SKM-1, Kasumi-1 (sensitive) versus HEL, KO52 (resistant)). Specifically, higher concentrations of BRD9 degrader are required to observe BRD9 degradation and growth inhibition for certain cell lines, such as HEL and KO52, as compared to other cell lines, such as MV4-11, SKM-1, and Kasumi-1. We anticipate that this may be related, at least in part, to differences in the degradation machinery in each AML cell type. Similarly, we observed a correlation between ALL and MM cell line sensitivity to BRD9 degrader treatment and BRD9 degradation, which suggests cell-based degradation mechanisms may be cell context-dependent and may explain why some transformed cells are more vulnerable to BRD9 degrader treatment than others.

Importantly, through RNA-seq analysis, we sought to identify BRD9-regulated signaling pathways among three heme malignancies to understand common and lineage-specific roles for BRD9. Pathway analysis revealed numerous uniquely deregulated genes in cells representing each of the three heme malignancies following BRD9 degradation. We also observed some common overlapping signaling pathways related to proliferation, immune response, inflammatory response, cell adhesion, and DNA repair/replication. This finding is consistent with deregulated signaling pathways in AML, but unexplored in the context of non-AML heme malignancies. These commonalities may be mechanistically related to the BRD9 dependence shared by these different heme malignancies and could be potentially exploited for therapy. For example, including a BRD9 degrader in drug combinations should be considered for clinical testing and may be effective in front-line induction of remission. Differentiation-inducing agents, such as decitabine and ATRA, are in fact strongly potentiated by BRD9 degrader and inhibitor treatment.

A role for BRD9 in the maintenance of MYC expression in AML has previously been reported for the mouse RN-2 cell line and the human NOMO-1 cell line [17]. We observed MYC to be downregulated in some cell lines, but not in others. In AML, whereas MYC downregulation correlated with BRD9 degradation in Kasumi-1 and MV4-11 cells, it did not for KO52, HEL and BRD9 degrader-sensitive SKM-1 cells. Similarly, MYC downregulation correlated with BRD9 degradation in H929 (MM) cells, but less so for the MM lines, MM.1S and U266. However, for the ALL cell line, RS4;11, which is inhibited by- BRD9 degraders, a MYC signature was not apparent following BRD9 degrader treatment. Our conclusion is that while BRD9 may play a role in maintenance of MYC expression in some acute leukemia and MM cells, it does not play a universal role in maintenance of MYC expression. Thus, further studies geared toward gaining a better understanding of the biological significance of a BRD9-MYC axis in heme malignancies are warranted.

AML has been treated using chemotherapy to induce remissions for decades, and despite advances in molecular characterization of primary and recurrent disease, new drug approvals have been few. Several recognized subtypes possess additional layers of heterogeneity that include cytogenetic factors and age of onset, which make it difficult to define unique drug targets as candidates that might induce long-term remissions such as BCR-ABL in chronic myeloid leukemia (CML). Combinations of drugs therefore are likely to endure as a mainstay for these patients. In consideration of genetic and phenotypic heterogeneity, we employed the use of a drug combination that could offer increased response rates. We find that modulating BRD9 potentiates the effects of numerous agents that are currently part of the standard treatment regimen for AML and other hematologic malignancies to induce differentiation and/or cytotoxic outcomes. Particularly robust effects were observed when combining targeted BRD9 degraders with venetoclax, decitabine, or ATRA. We also show that primary AML cells that are relatively resistant to BRD9 degrader treatment as a single agent are killed to a greater extent when the BRD9 degrader is combined with standard chemotherapy. Taken together, our findings support further development of therapeutic targeting of BRD9, alone or combined with other agents, as a novel strategy for acute leukemias and MM.

The studies reported herein reveal a BRD9 dependency in acute leukemia and MM and highlight its importance for cell growth and signaling in these diseases. Our findings also unveil the robust synergizing potential of selective BRD9 targeting in hematopoietic malignancies.

## Supplementary information

Supplementary Figure Legends- clean

Supplementary Figures 1-12

Supplementary Table 1-Primary Patient Information

Supplementary_Materials and Methods

## Data Availability

The structures of the compounds, EA-89 and QA-68, have been deposited at the public PDB-database (https://www.rcsb.org) and can be accessed by the PDB-code (8A7I). RNA-Seq datasets have been deposited in Sequence Read Archive (SRA) and can be accessed by Project accession ID (PRJNA850940). Information is accessible using the following link: http://www.ncbi.nlm.nih.gov/bioproject/850940
