# Supplementary material for: BRD9 degraders as chemosensitizers in acute leukemia and multiple myeloma
Source: Blood Cancer J. 2022 Jul 19;12(7):110. doi: 10.1038/s41408-022-00704-7 (PMC9296512; doi:10.1038/s41408-022-00704-7)
Supplement: Supplementary file 1 — Supplementary Figure Legends- clean [file 41408_2022_704_MOESM1_ESM.docx]

**Supplementary Figure Legends**

**Supplementary Figure 1. BRD9 degrader versus BRD9 inhibitor inhibition of AML cell line growth.** (A-E) Proliferation assays: AML cell lines treated for 6 days with EA-89, QA-68, or dBRD9-A at the indicated concentrations. (F-J) Effects of QA-68 on growth over time of AML cell lines.

**Supplementary Figure 2. Effects of EA-89 treatment on AML cell line proliferation, differentiation, and cell cycle progression.** (A) Proliferation assays: AML cell lines treated with EA-89 for 6 days. (B) Differentiation study: SKM-1 cells were treated with EA-89 for 4 days. (C) Effects of EA-89 on cell cycle progression in SKM-1. Treatments were carried out for 6 days for cell cycle studies.

**Supplementary Figure 3(A). Effects of BRD9 degrader versus BRD9 inhibitor treatment on differentiation of SKM-1 and MV4-11 cells.** Treatment of SKM-1 cells for 6 days leads to greater induction of differentiation by BRD9 degrader, QA-68, than the BRD9 inhibitor, EA-89.

**Supplementary Figure 3(B). Effects of BRD9 degrader versus BRD9 inhibitor treatment on differentiation of SKM-1 and MV4-11 cells.** Treatment of SKM-1 cells for 6 days with dBRD9-A leads to induction of differentiation. Unstained, CD11b and CD14 results are shown as controls.

**Supplementary Figure 4. Effects of BRD9 KD in MV4-11 cells.** (A) Proliferation assays. The antiproliferative effects of BRD9 KD in MV4-11 cells, using plko.1 GFP short hairpins against BRD9 versus non-target hairpins, are compared. Y-axis shows percent GFP positive cells. (B) Assessment of BRD9 mRNA levels in non-target KD controls and BRD9 KD lines.

**Supplementary Figure 5. Effects of BRD9 targeting on B-ALL cell line growth, cell cycle progression, and apoptosis.** (A-E) Proliferation assays: NALM6 cells (A), SEM cells (B), RS4;11 cells (C) and SEMK2 cells (D) treated with QA-68 or dBRD9-A for 6 days. (E) Proliferation assay: SEMK2 cells treated with QA-68 or EA-89 for 6 days. (Right panels): Effects of dBRD9-A or QA-68 on BRD9 and c-Myc expression following 24 hr treatment. (F) Proliferation assays: SEM, NALM6, and RS4;11 cells treated with EA-89 for 6 days.

**Supplementary Figure 6(A-D). Effects of BRD9 targeting on ALL cell line growth: B-ALL cells.** Treatment of REH, SUP-B15, 697, and RCH-ACV cells, with EA-89, QA-68, or dBRD9-A.

**Supplementary Figure 6(E-L). Effects of BRD9 targeting on ALL cell line growth: T-ALL cells.** (E) Proliferation assays: PF-382 cells treated with QA-68 or dBRD9-A or EA-89 for 6 days. Data are shown as CellTiter Glo absorbance values. Proliferation assays: Jurkat cells (F), LOUCY cells (G), CCRF-CEM cells (H), DND-41 (I), HPB-ALL (J), KOPT-K1 (K), and MOLT4 (L) treated with QA-68, EA-89 or dBRD9-A for 6 days. Data for B-F are shown as percent of DMSO-treated controls. Data for K are shown as CellTiter Glo absorbance values.

**Supplementary Figure 7. Potentiation of daunorubicin by BRD9 degrader treatment and effects of BRD9 degraders and inhibitors on normal bone marrow cells.** (A-B) Proliferation assays: RS4;11 (C) or primary ALL3 (D) cells treated with daunorubicin alone, QA-68 at a fixed concentration alone, or a combination of both agents. (C) Proliferation assays: QA-68 and EA-89 treatment of normal bone marrow (sample #3) (in the absence and presence of cytokines as indicated) for 6 days. MV4-11 cell lines were tested as a positive control.

**Supplementary Figure 8. Effects of QA-68 on BRD4 and BRD9 expression in AML and MM cells.** (A) 24-hour QA-68 (1000 nM) treatment of SKM-1 and H929 cells, versus HEK293 (as a non-heme malignancy control). (B) Effects of 24-hour and 48-hour continuous QA-68 (10 nM) treatment, respectively, of MV4-11 on BRD4 and BRD9 protein expression, compared with 24-hour QA-68 treatment followed by 24-hour washout of QA-68.

**Supplementary Figure 9. Effects of BRD9 inhibition and degradation on MM cell line growth; BRD9 degrader potentiation of pomalidomide against MM cells.** (A) Proliferation assays: H929 cells treated with EA89, QA-68, or dBRD9-A for 6 days. Data are shown as percent of DMSO-treated controls. (B) Proliferation assay: MM.1S cells treated with QA-68 or dBRD9A for 6 days. Data are presented as CellTiter Glo absorbance values. (C) Proliferation assay: H929 or MM.1S treated with EA-89 for 6 days. Data are presented as percent of DMSO-treated controls. (D) Effects of dBRD9-A on BRD9 expression in H929 and MM.1S cells. (E) Proliferation assays: U266 cells treated with EA-89, QA-68 or dBRD9A for 6 days (data are shown as CellTiter Glo absorbance values). (F) Effects of QA-68 or dBRD9-A on BRD9 expression following 24 hr treatment. (G) Treatment of 8226 with EA-89, QA-68 or dBRD9-A for 6 days (data are shown as CellTiter Glo absorbance values). (H) Treatment of MM.1S cells for 6 days with QA-68 alone, pomalidomide alone, or a combination. (I) Treatment of H929 cells for 6 days with dBRD9-A alone, pomalidomide alone, or a combination.

**Supplementary Figure 10. Proliferation studies investigating the effects of BRD9 degrader treatment alone versus combination with ATRA against AML cells.**

**Supplementary Figure 11. Targeted inhibition or degradation of BRD9 positively combines with chemotherapy agents and targeted inhibitors to induce differentiation of AML cells.** Effects of QA-68 or EA-89 alone and combined with other agents on differentiation status of SKM-1 cells. SKM-1 cells were treated for 6 days.

**Supplementary Figure 12 (A-C). RNA-seq analysis of ALL, AML and MM cells treated with BRD9 degraders.** (A) Venn Diagram displaying the number of unique and conserved DEGS after QA-68 or dBRD9-A treatment in MV4-11. (B) Gene Set Enrichment Analysis of dBRD9-A treatment induced differentially expressed genes (DEGs) in MV4-11. (C) qPCR validation of genes deregulated following BRD9 degrader treatment of MV4-11. DEGs are defined by Benjamini & Hochberg FDR <0.05 & |FC|>1.5 between QA-68 and DMSO treatments.

**Supplementary Figure 12 (D-E). RNA-seq analysis of ALL, AML and MM cells treated with BRD9 degraders.** (D-E) qPCR validation of genes deregulated following BRD9 degrader treatment of H929 (D) and RS4;11 (E) cells. DEGs are defined by Benjamini & Hochberg FDR <0.05 & |FC|>1.5 between QA-68 and DMSO treatments. Specifically, of the downregulated genes common to representative sensitive models of AML, ALL and MM, significant were those that are involved in cell division (TERT, CDC6, E2F1, DDIAS, CDC25A, CDC45, PKMYT1), DNA replication (RRM2, RECQL4, ORC6) and DNA repair (RAD51, PCNA, FEN1) among others. Similarly of the upregulated genes common to representative sensitive cells, identified genes were identified as implicated in the immune response (C5, TLR4, RSAD2, OAS3, CX3CR1, CSF1R, OAS1), inflammatory response (ALOX5AP, CCL2), cell division (KIFC3), monocyte differentiation (CSF1R, PDE1B, ABCD2) and transcriptional regulation (BATF2, EPAS1, ZFHX2, TBX19, ZNF467, ZNF425, SATB1, ZBED6).
