# Supplementary material for: BRD9 degraders as chemosensitizers in acute leukemia and multiple myeloma
Source: Blood Cancer J. 2022 Jul 19;12(7):110. doi: 10.1038/s41408-022-00704-7 (PMC9296512; doi:10.1038/s41408-022-00704-7)
Supplement: Supplementary file 2 — Supplementary Figures 1-12 [file 41408_2022_704_MOESM2_ESM.pdf]

Supplementary Figure 1

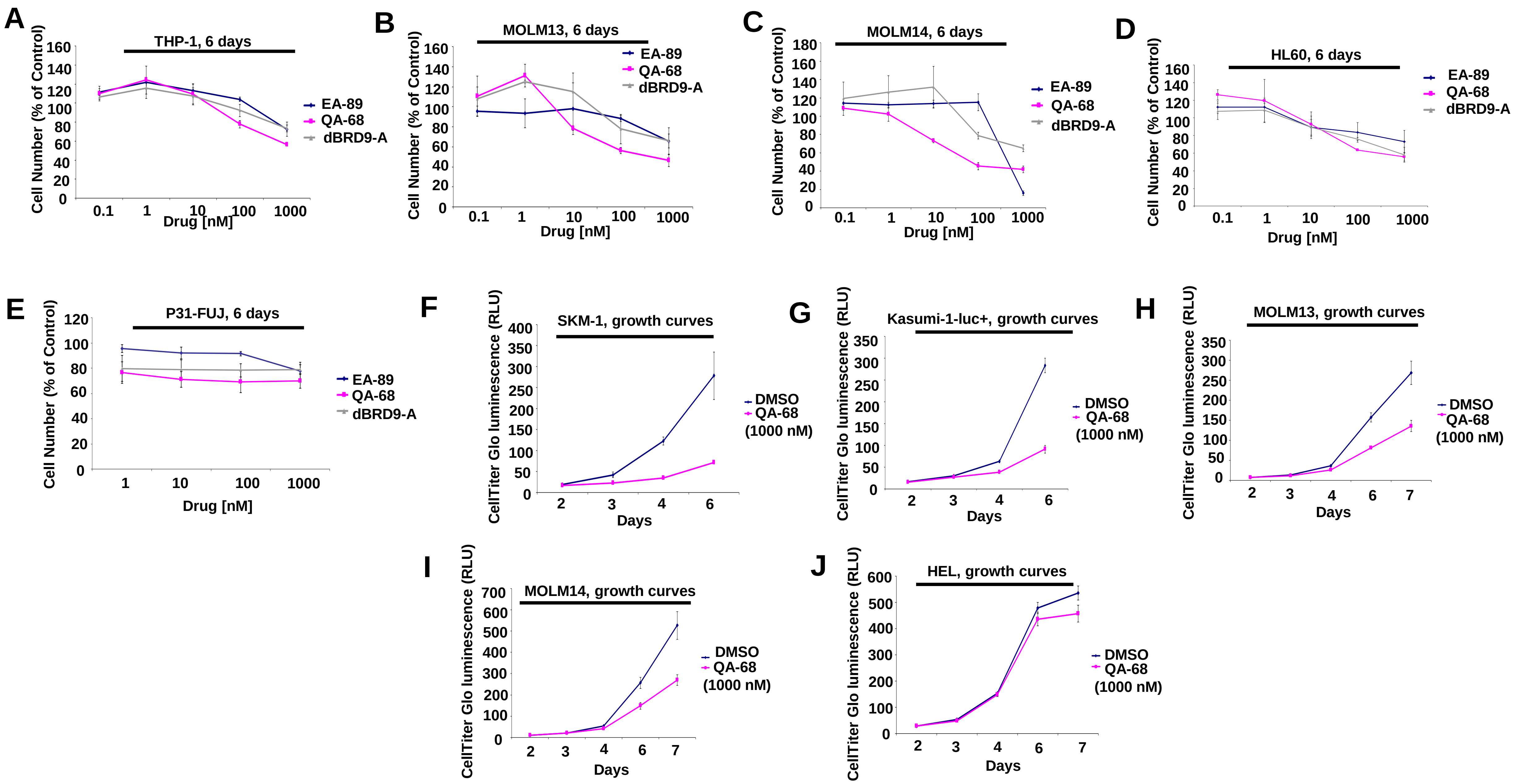

Supplementary Figure 2

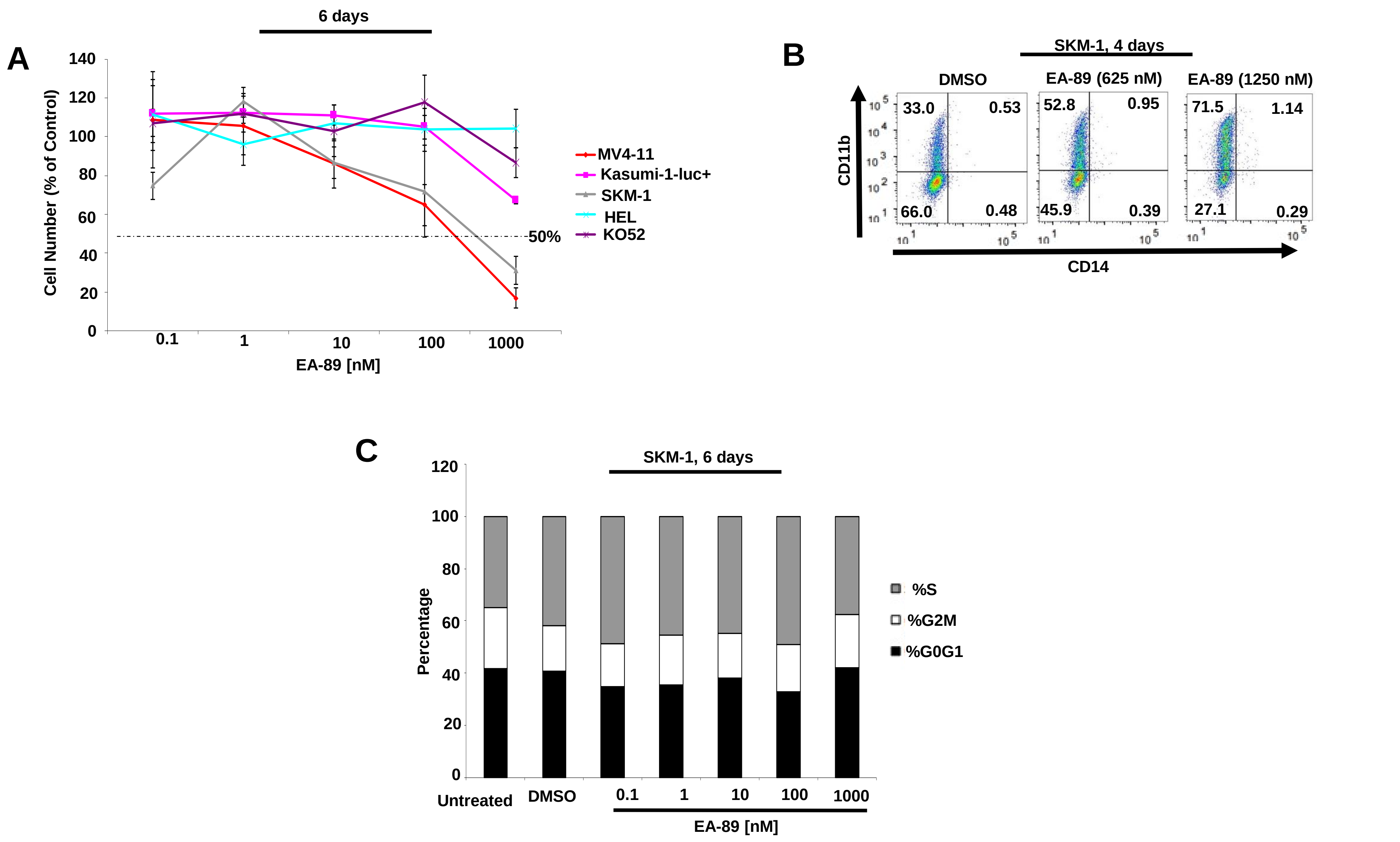

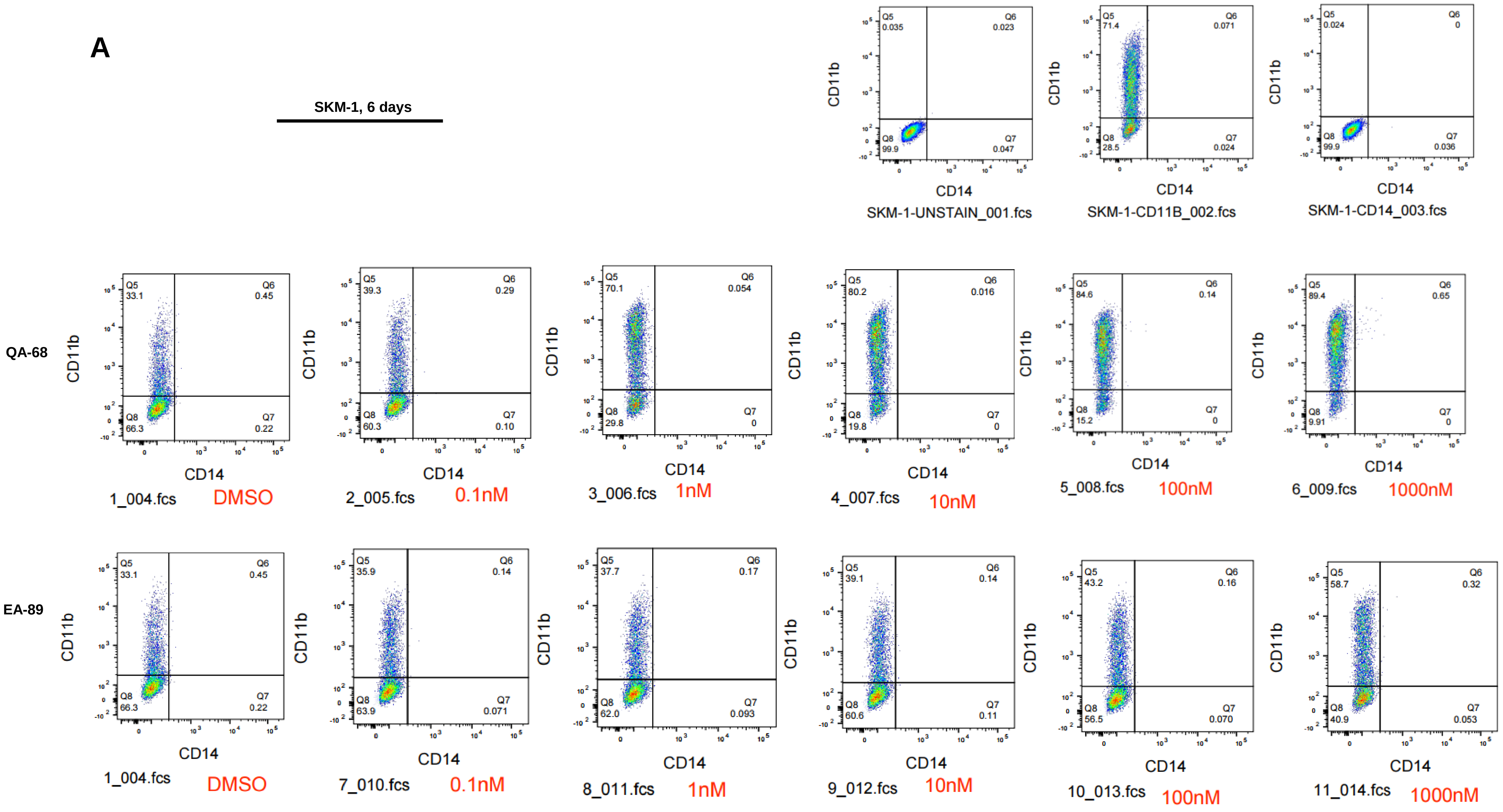

Supplementary Figure 3(A)

B

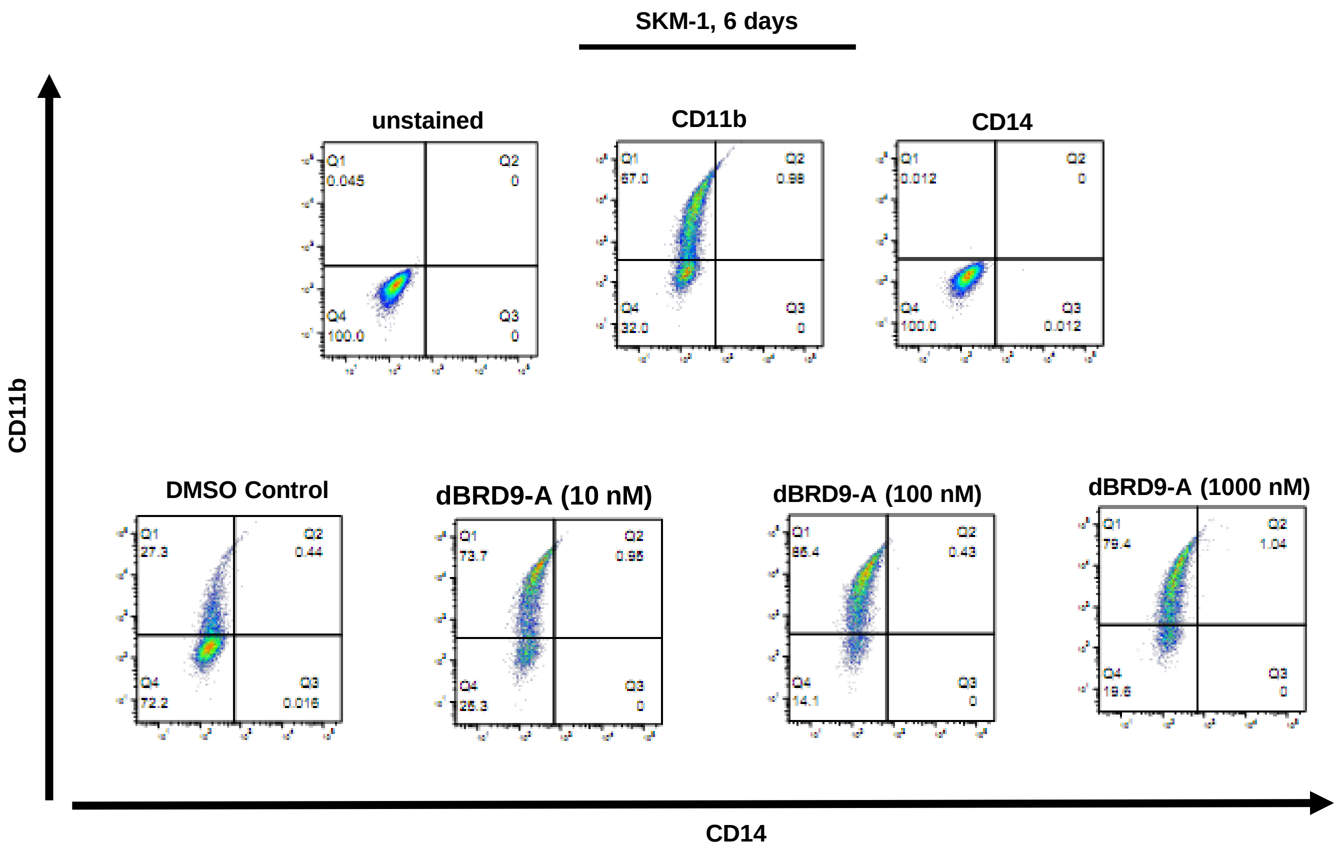

Supplementary Figure 3(B)

A

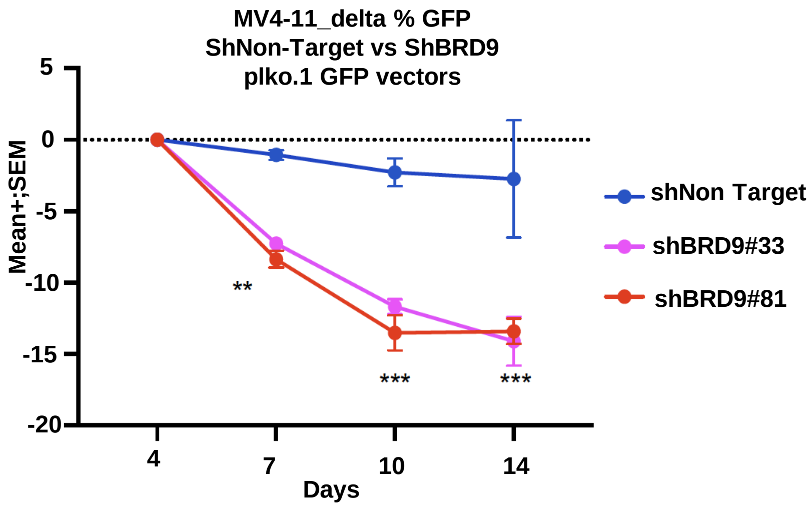

B

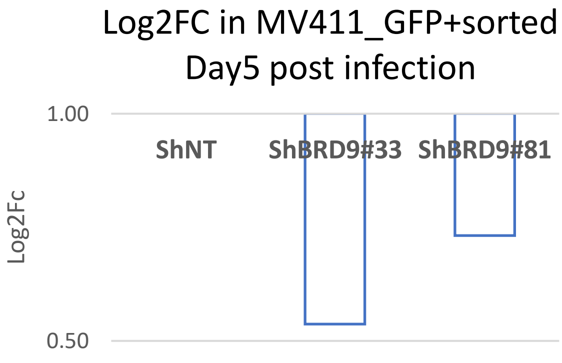

Supplementary Figure 4

Supplementary Figure 5

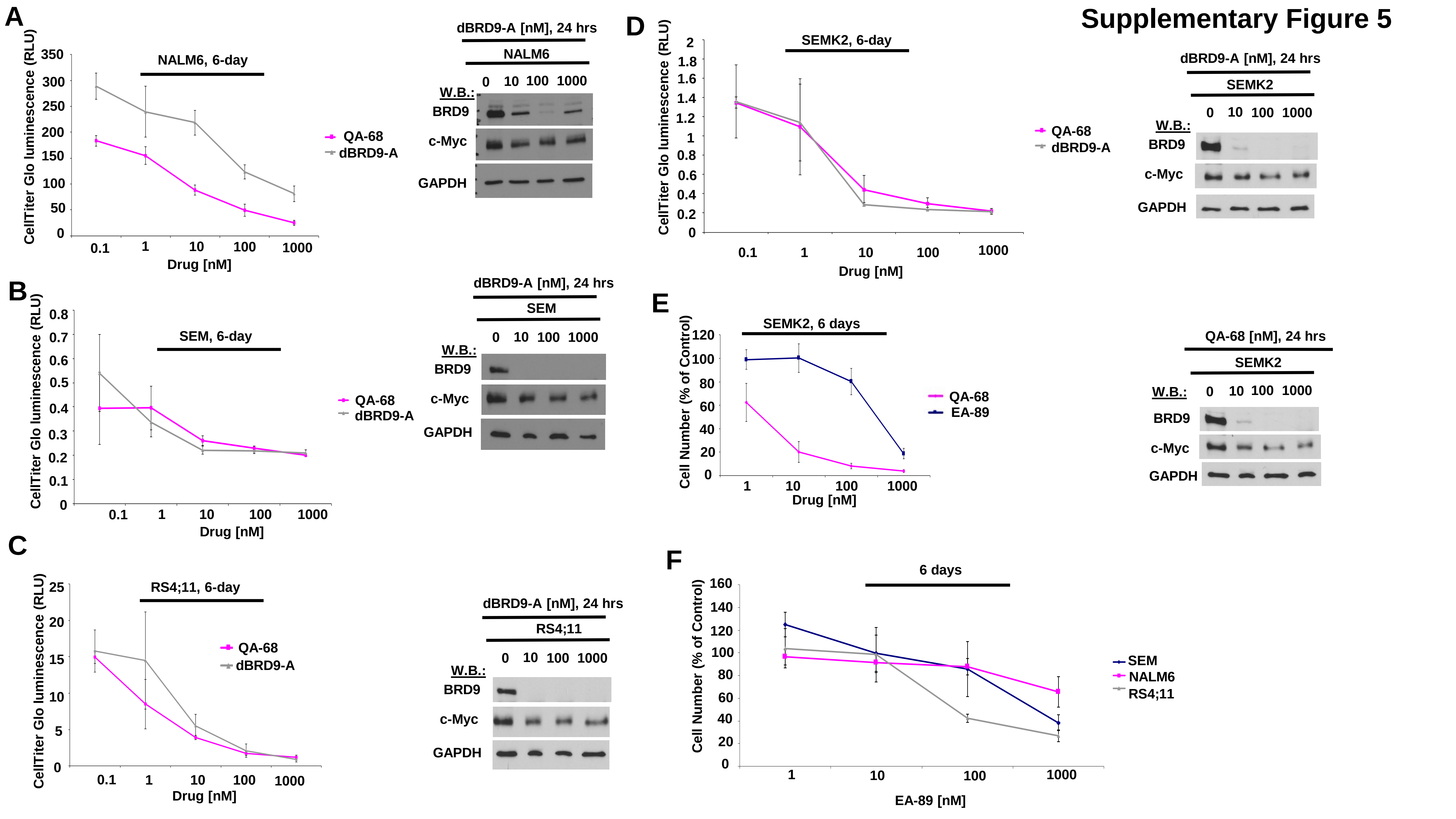

Supplementary Figure 6 (A-D)

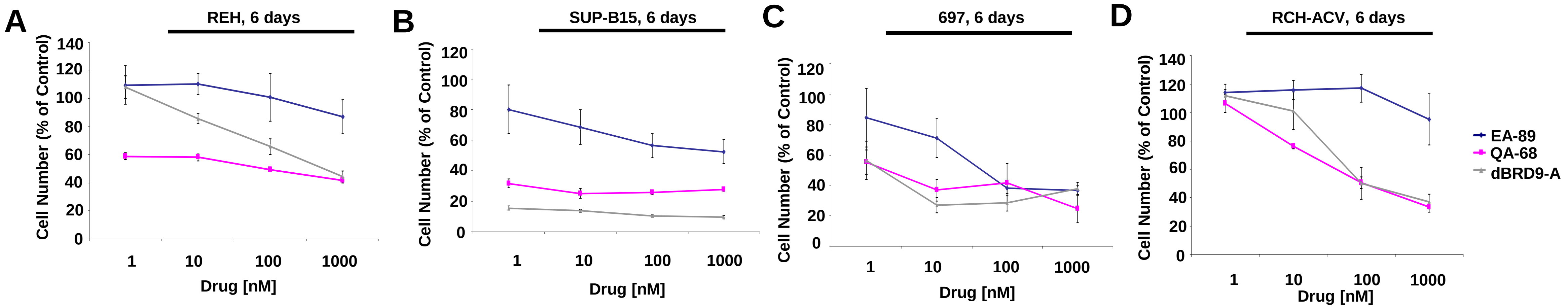

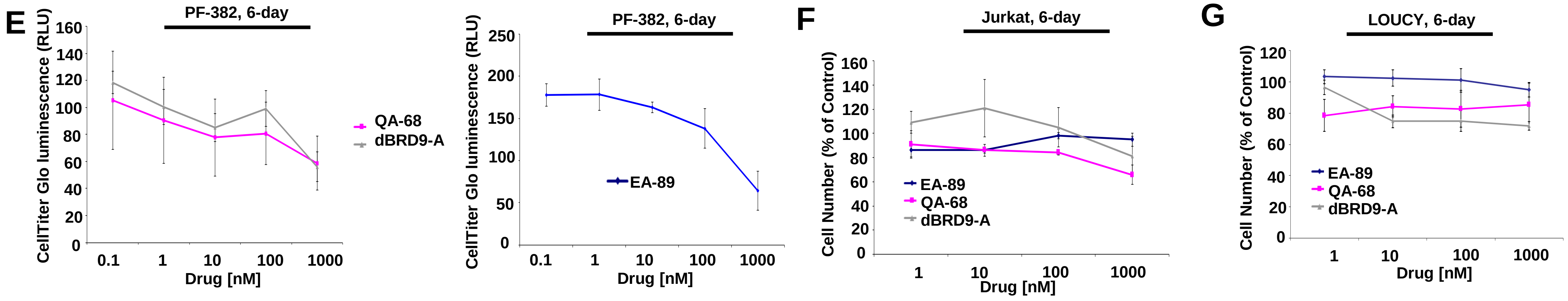

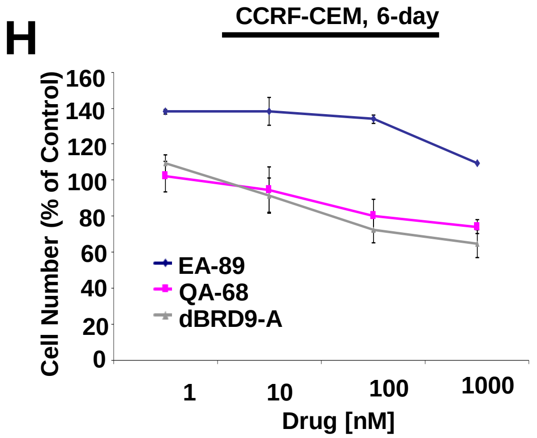

Supplementary Figure 6 (E-L)

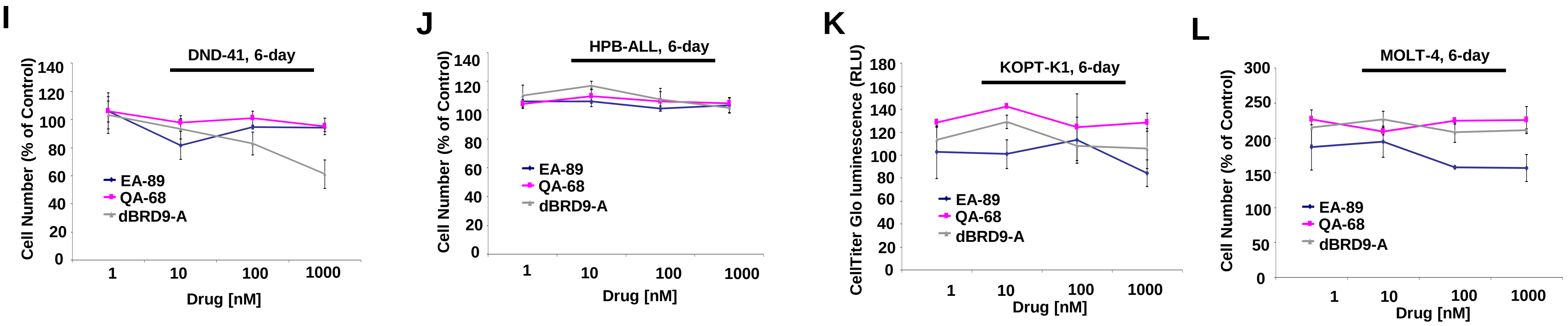

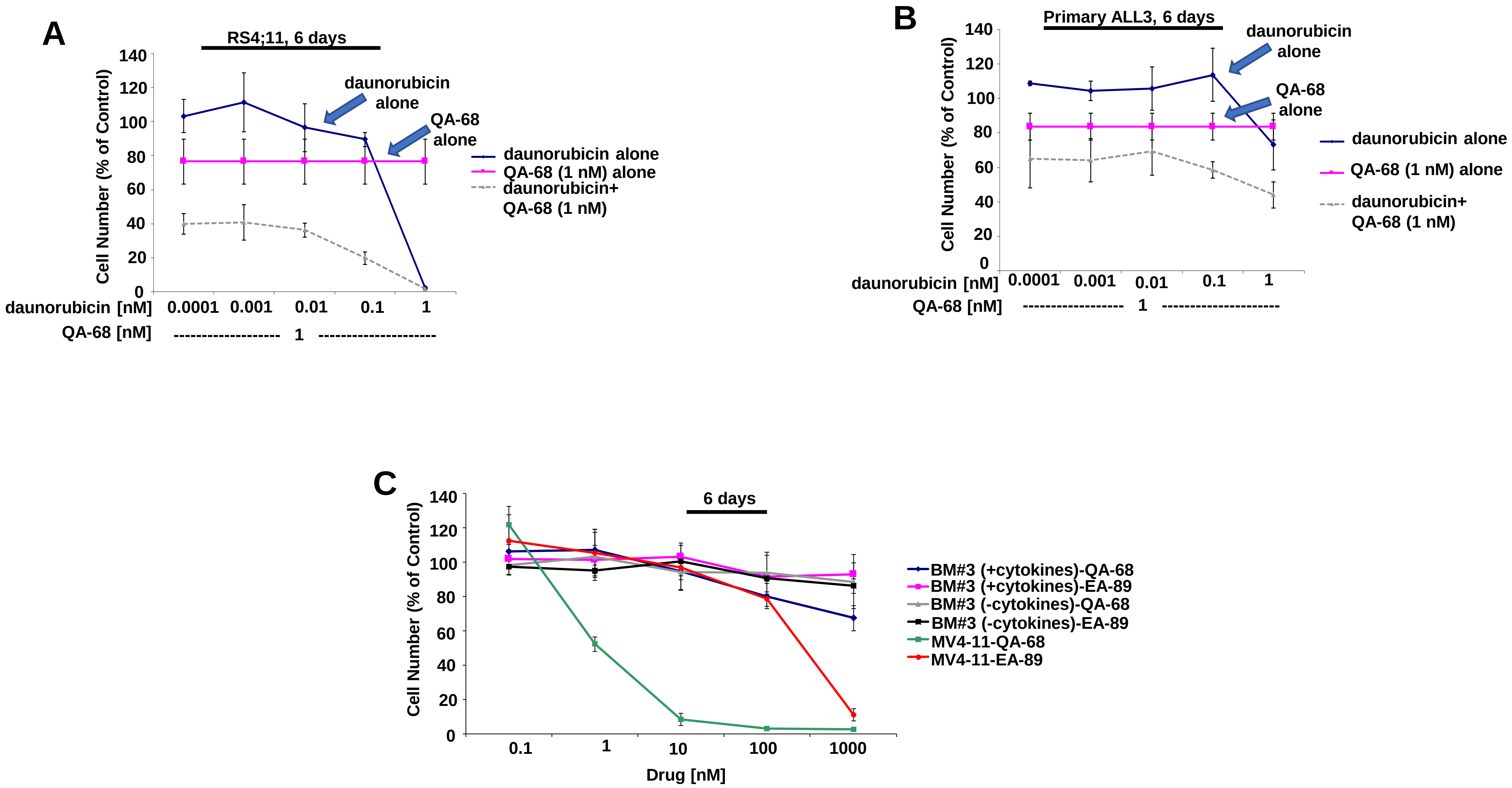

Supplementary Figure 7

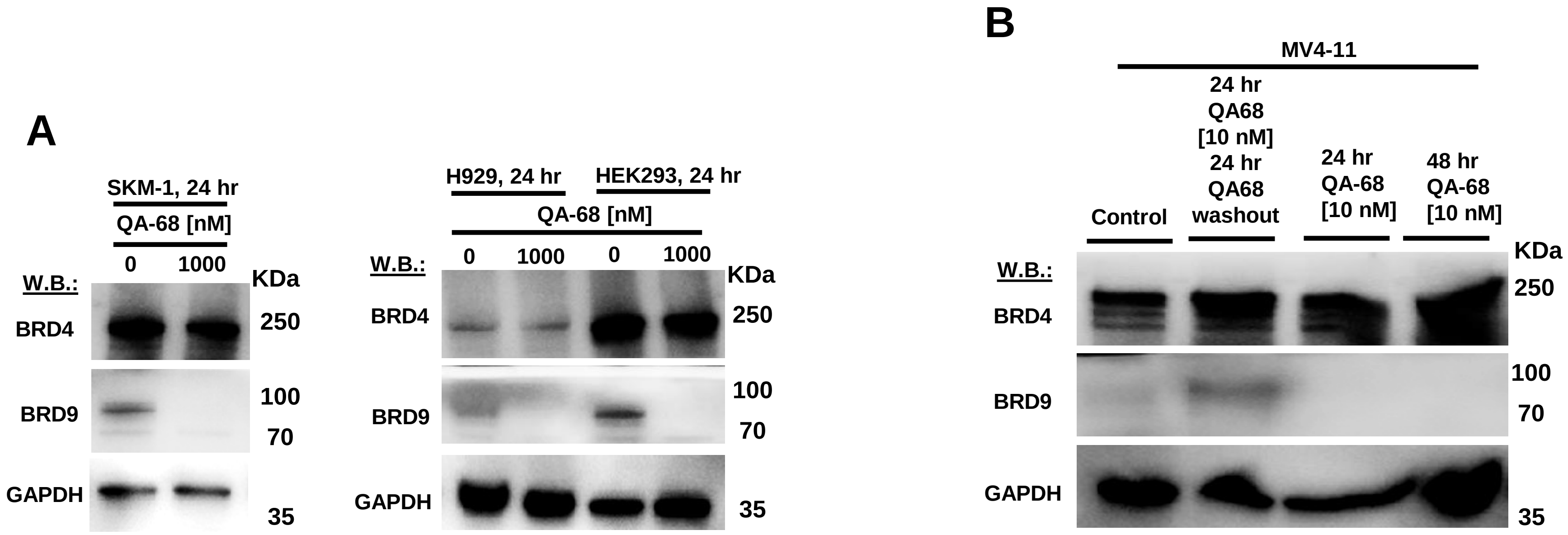

Supplementary Figure 8

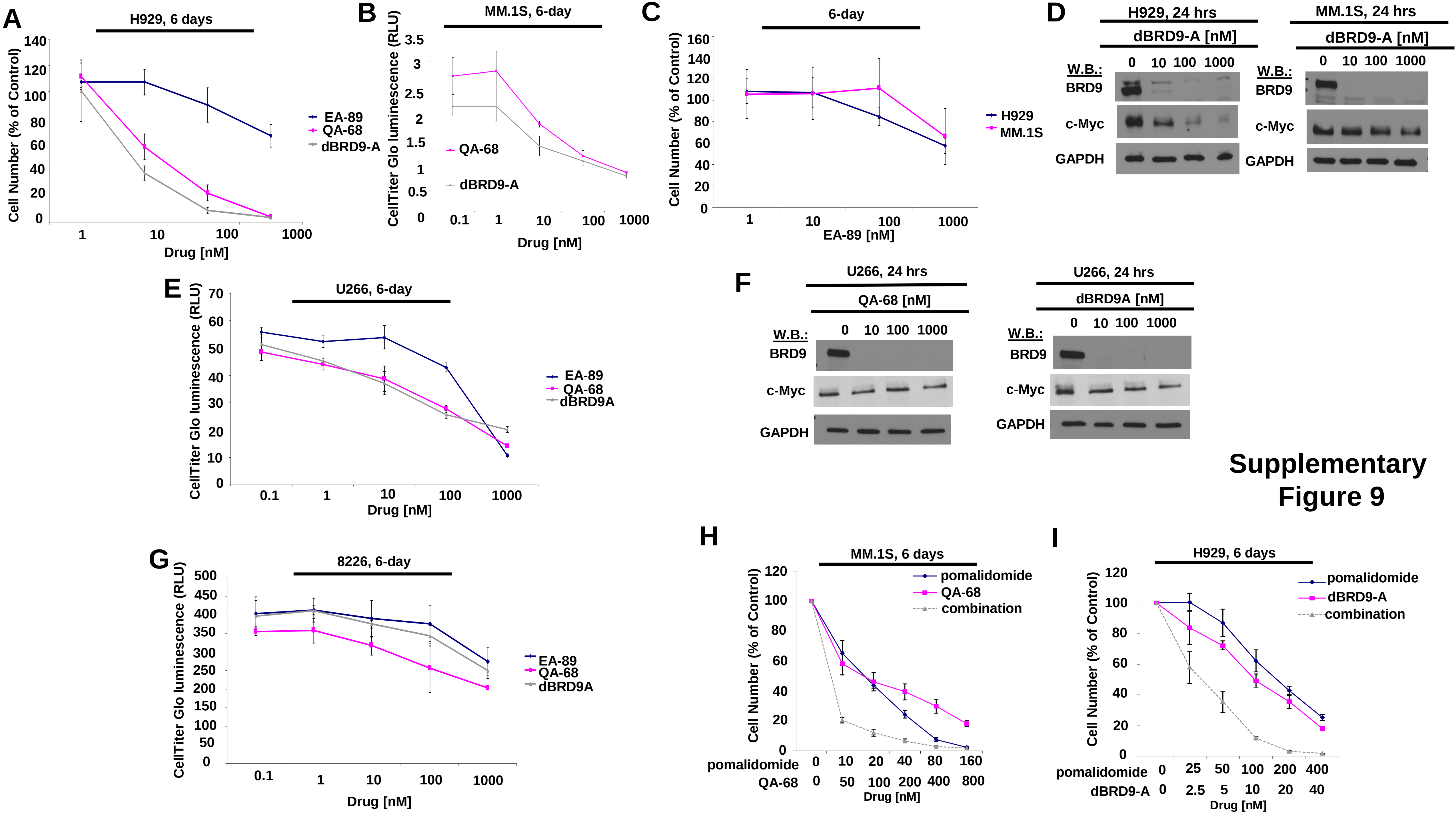

Supplementary Figure 9

Supplementary Figure 10

A

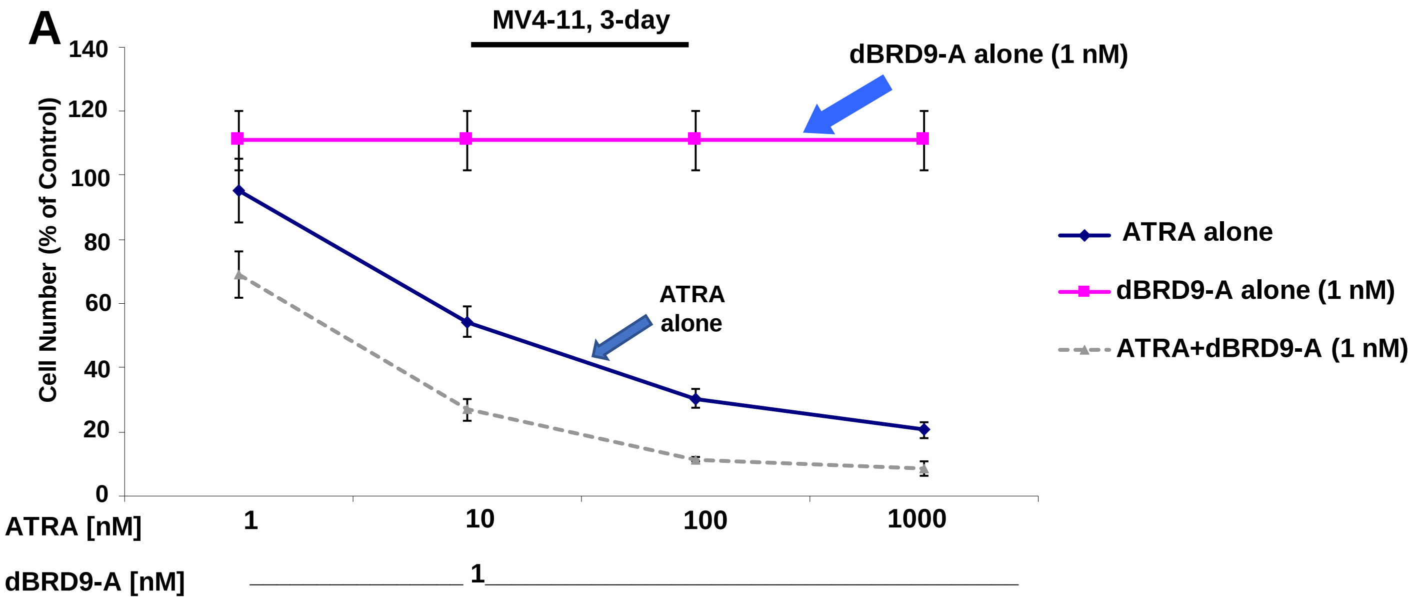

B

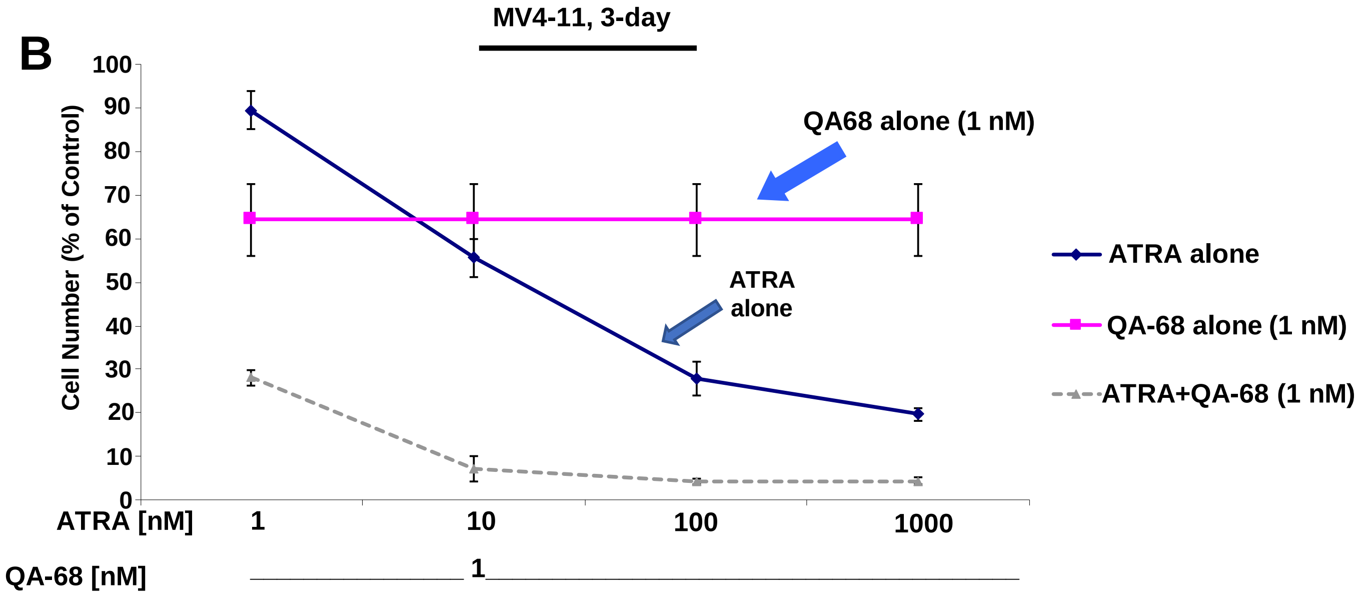

SKM-1

	Treatment	CD11b
	DMSO	38.7
	QA68 (0.1 nM)	45.8
	decitabine (25 nM)	64.4
**Drug Combination**	QA68 (0.1 nM)+decitabine (25 nM)	73.4
	DMSO	38.7
	QA68 (0.1 nM)	45.8
	5-azacytidine (25 nM)	43.8
**Drug Combination**	QA68 (0.1 nM)+5-azacytidine (25 nM)	55.1
	DMSO	38.7
	QA68 (1 nM)	84.5
	venetoclax (625 nM)	83.6
**Drug Combination**	QA68 (1 nM)+venetoclax (625 nM)	97.1
	DMSO	38.7
	EA89 (1000 nM)	65.7
	decitabine (25 nM)	64.4
**Drug Combination**	EA89 (1000 nM)+decitabine (25 nM)	83.4
	DMSO	38.7
	EA89 (1000 nM)	65.7
	5-azacytidine (25 nM)	43.8
**Drug Combination**	EA89 (1000 nM)+5-azacytidine (25 nM)	61.1
	DMSO	38.7
	EA89 (1000 nM)	65.7
	venetoclax (625 nM)	83.6
**Drug Combination**	EA89 (1000 nM)+venetoclax (625 nM)	92.9

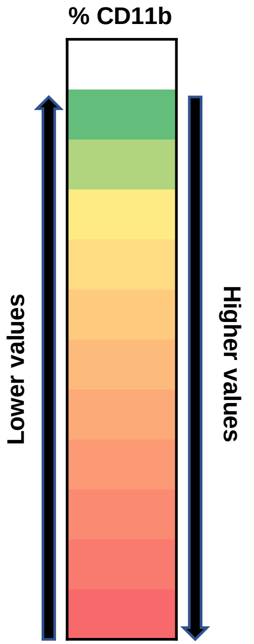

Supplementary Figure 11

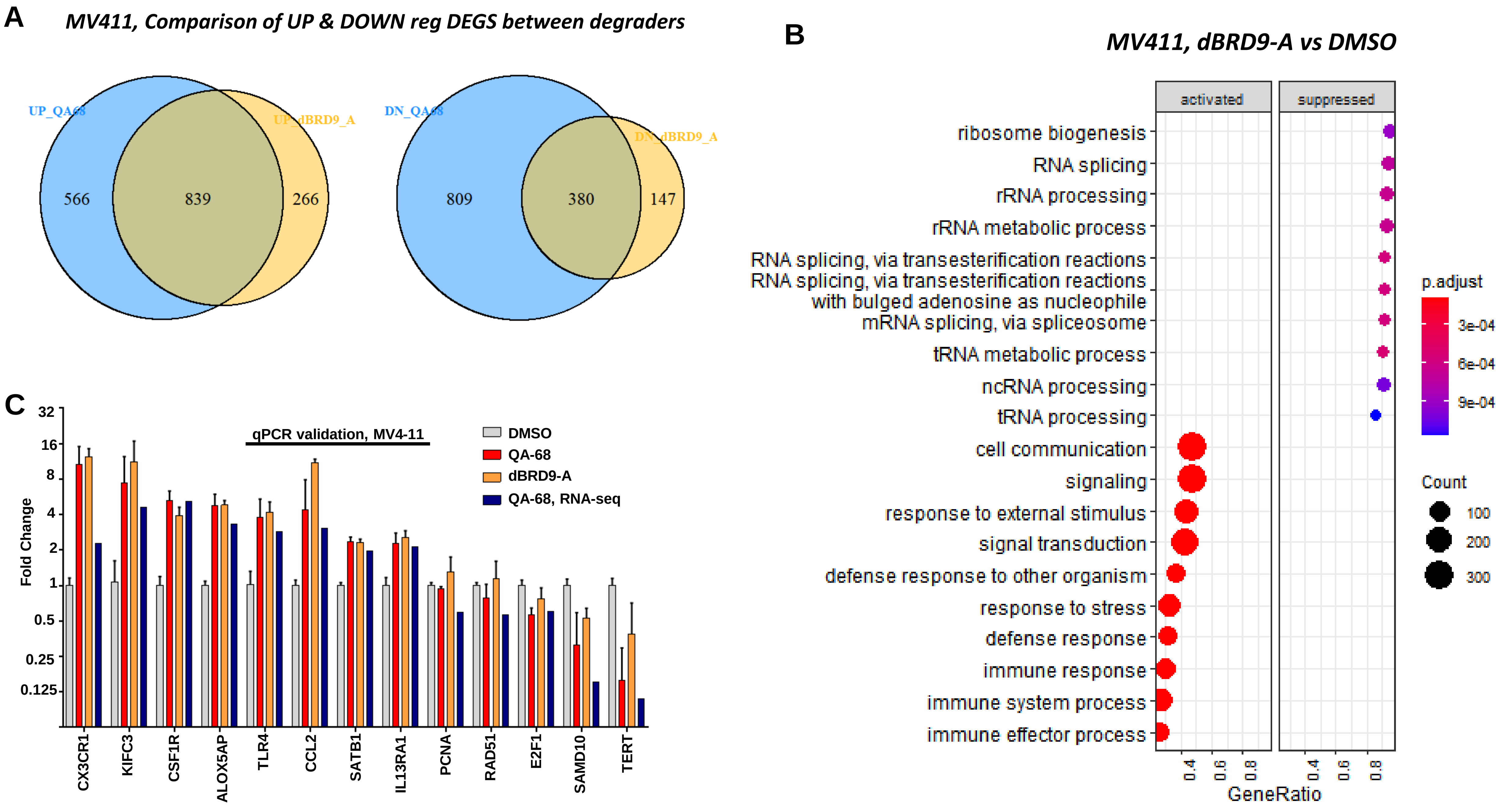

Supplementary Figure 12 (A-C)

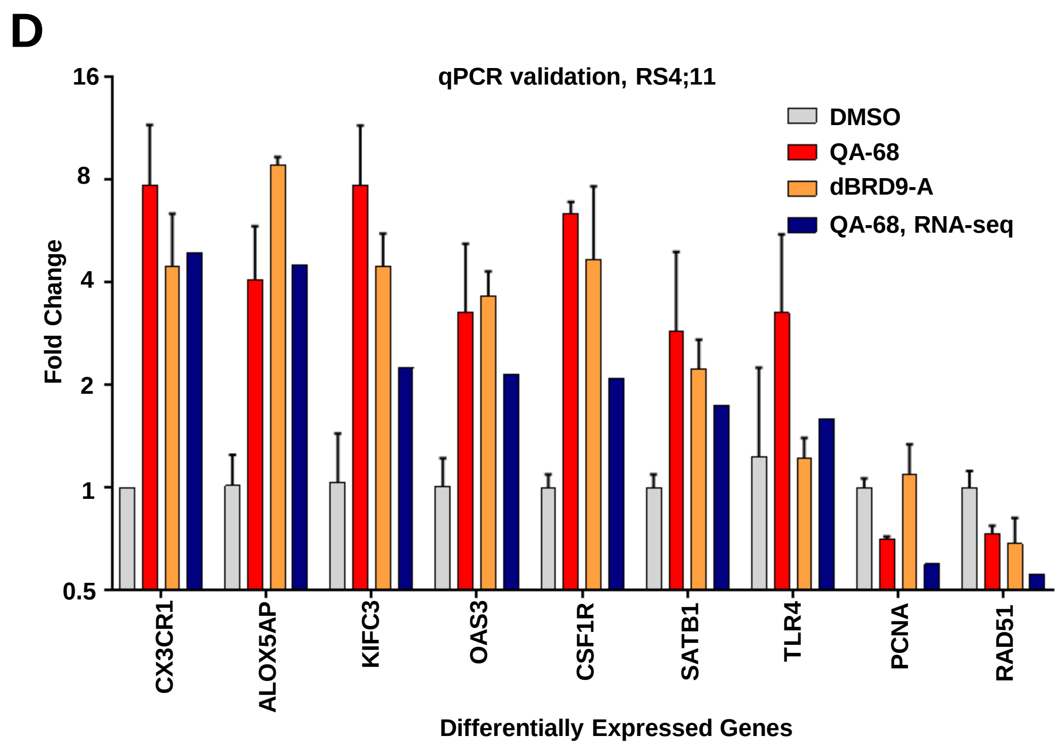

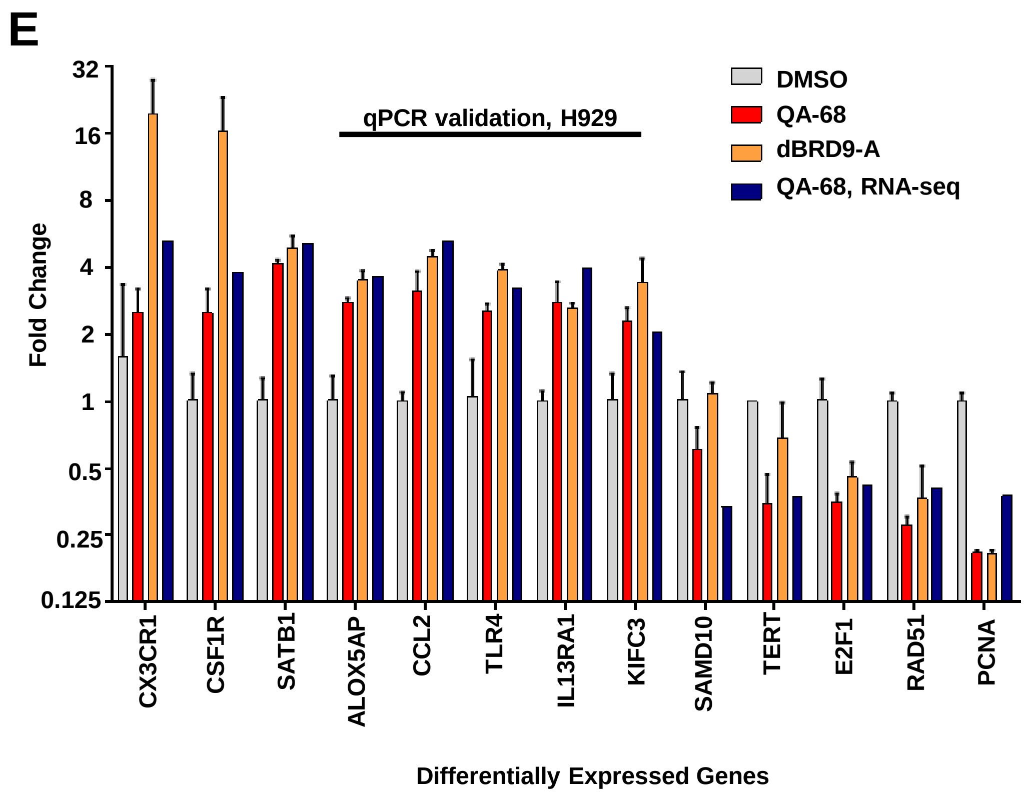

Supplementary Figure 12 (D-E)
