# Supplementary material for: BRD9 degraders as chemosensitizers in acute leukemia and multiple myeloma
Source: Blood Cancer J. 2022 Jul 19;12(7):110. doi: 10.1038/s41408-022-00704-7 (PMC9296512; doi:10.1038/s41408-022-00704-7)
Supplement: Supplementary file 3 — Supplementary Table 1-Primary Patient Information [file 41408_2022_704_MOESM3_ESM.doc]

| **Primary AML or ALL patient sample**  **(date of patient sample vial information acquired;**  **date of patient sample vial tested in study)** | **Gender/Age** | | **% blasts** | **Cytogenetics** | **Mutations** |
| --- | --- | --- | --- | --- | --- |
| ALL1  acute lymphoid leukemia  (12/28/2017) | Male/33  (deceased 2018) | | ND | 46,XY,add(5) (q?31),del(12) (p12),?del(17) (p?11.2p?13) [cp5]/46,idem,?del(2)(q?37) [cp4]/46,XY [cp11] | ND |
| ALL3  acute lymphoid leukemia in remission (disorder)  Unlisted pathology: The overall morphologic and immunophenotypic findings are consistent with PERSISTENT INVOLVEMENT by the patient's known B LYMPHOBLASTIC LEUKEMIA. Legacy Pathology: ALL - No classification  (6/20/2012) | Male/44 | | 56  (6/20/2012) | 46,X,-Y,del(9)(q13),add(10)(q24),t (16;22) (q11;q13),+22 [11]/46,XY [9]. ish t(16;22) (Tel16pter+, CBFB;CBFB+,Tel16qter+) | ND |
| AML4  secondary acute myeloid leukemia  (7/20/2018; 9/17/2018) | Female/  49 | | 98  (7/20/2018)  81 (9/17/2018) | 46,XX,del(13) (q12q22)[12]/46,XX[8] | | IDH1 NM_005896 c.394C>A p.R132S  in 44.3% of 926 reads | | --- | | NPM1 NM_002520 c.861_862insTGCA  p.W288fs* >9 - in 46.5% of 271 reads | | NRAS NM_002524 c.38G>A p.G13D –  in 47.9% of 928 reads | | RIT1 NM_006912 c.229G>A p.A77T –  in 1.0% of 1244 reads* | |
| AML14  acute myeloid leukemia- no classification; the overall findings are of a hypercellular marrow with extensive involvement by patient acute myeloid leukemia  (7/17/2015) | | Female/44  (deceased) | 55  (7/17/2015) | 45, XX, t(3;3) (q21;q26),-7[20] | ND |
| AML15 (PBMC) | | Female/69 |  | 47, XX, +19, der (19)t(1;19) (q21;q13.3)[14]/49,idem,t(8;14) (q12;q31), +der(8)t(8;14) x2,del(11) (q13q33)[cp6]  Each of 20 metaphases from this unstimulated bone marrow specimen has a derivative (1;19) translocation, resulting in partial triplication of 1q. A second clone has the same aberration plus an (8;14) translocation. By report, both of these aberrations were observed in a previous specimen processed at an outside laboratory. In the current specimen, the second clone also has two extra copies of the derivative 8 and a deletion of 11q, suggesting clonal evolution and consistent with a persistent or recurrent myeloid disorder. | CALR      c.1154_1155insTTGTC (p.K385Nfs*47) 45.9% VAF (1007x consensus coverage) TET2      c.2421delG (p.E808Kfs*5) 1.7% VAF (1174x consensus coverage)# TET2      c.4396C>T (p.Q1466*) 1.4% VAF (1290x consensus coverage)#  #These variants are located on different amplicons; therefore, phasing (cis versus trans) cannot be assessed.  *This test is designed for somatic analysis of variants and may provide information about the germline. It cannot distinguish between somatic and germline variants.   Potential Germline (with possible secondary somatic) Variants (SBDS, TERC, TERT, DKC1, and DDX41 ONLY)*:  Potential Germline Pathogenic Single Nucleotide Variants and Small Insertions/Deletions: None Detected.  Potential Germline Variants of Unknown Signification (VUS):  DKC1      c.838A>C (p.S280R) 38.4% VAF (1716x consensus coverage)#   #Of note, the listed variant in DKC1 has been noted at a very low frequency in the general population in SNP databases.  *These genes have been associated with germline disease.  Copy Number Analysis*:  Read count analysis shows:  1 copy deletion DNMT3A (on 2p); POSSIBLE 1 copy deletion ATM (on 11q); gain JAK3 (on 19p)  IKZF1 deletion: not detected ERG deletion: not detected KMT2A(MLL)-PTD: not detected  *This test assesses only autosomal chromosomal copy number changes.  FLT3-ITD Analysis: None Detected. |
| AML16  (BM) | | Female/89 | 33  (8/16/2021) | 46,XX,add(16) (p13.?2),del(16)(p1?2)[18]/46,XX[1].ish add(16)(MYH11+,CBFB+), del(16) (MYH11, CBFB+) [3].nuc ish (MYH11x1,CBFBx2)[46/100]  18 of 19 metaphases from this unstimulated peripheral blood specimen contained two abnormal copies of chromosome 16, one with a deletion on the p-arm and the other with additional material on the p-arm, which were seen in a previous sample by report. Therefore, these findings are consistent with a recurrent or persistent myeloid disorder.  FISH analysis on abnormal metaphases and interphase nuclei did NOT demonstrate CBFB-MYH11 rearrangement, but demonstrated loss of MYH11 locus on the del(16p). | ASXL1     c.1926_1927insG (p.G646Wfs*12) 3.1% VAF (1656x consensus coverage) *NEW* BCOR      c.3205G>T (p.E1069*) 1% VAF (1869x consensus coverage)# *NEW* GATA2     c.913C>G (p.L305V) 34.8% VAF (1722x consensus coverage)^ IKZF1     c.637C>T (p.R213*) 5.4% VAF (1266x consensus coverage) *NEW* PHF6      c.1003_1004insA (p.R336Efs*3) 17.8% VAF (129x consensus coverage) *NEW* SRSF2     c.284C>A (p.P95H) 36% VAF (1457x consensus coverage) TET2      c.1720C>T (p.Q574*) 24.9% VAF (2009x consensus coverage)  #This variant is identified at a variant allele fraction lower than the validated threshold for calling a new variant and should be viewed with caution.   ^This GATA2 variant is located in a region near other pathogenic variants and tracks with the pathogenic SRSF2 mutation, so it is now annotated as a pathogenic variant  IKZF1 deletion: not detected ERG deletion: not detected KMT2A(MLL)-PTD: not detected  *This test assesses only autosomal chromosomal copy number changes.  FLT3-ITD Analysis: None Detected. |

| **Primary MM Patient Sample** | **Status** | **Date** | | **age** | **gender** | **diagnosis** | **Subtype** | **% Plasma cell** | **% Blast cells** | **Cytogenetics** |  |
| --- | --- | --- | --- | --- | --- | --- | --- | --- | --- | --- | --- |
| 1 | ND | 3/29/2021 | | 57 | M | MM | IgA L | 12 | 3 | Tri 1q, Tetra 11q, Tri 11q, Tri 17  No translocations |  |
| 2 | RR | 6/7/2019 | | 73 | M | MM | IgG K | 35 | 0 | Tri 1q, Del 1p, Tetra 14q, Penta 14q, Mono 13, Mono 16q  No translocations |  |
|  | | |  | | | | | | | | |
| 3 | ND | 6/3/2021 | | 79 | M | MM | IgA K | 32 | 0 | Teta 1p, Tetra 1q, Octo 1q, Mono 13, Tri 14q  No translocations |  |

**Supplementary Table 1. Primary patient sample characteristics.**
