# Supplementary material for: BRD9 degraders as chemosensitizers in acute leukemia and multiple myeloma
Source: Blood Cancer J. 2022 Jul 19;12(7):110. doi: 10.1038/s41408-022-00704-7 (PMC9296512; doi:10.1038/s41408-022-00704-7)
Supplement: Supplementary file 4 — Supplementary_Materials and Methods [file 41408_2022_704_MOESM4_ESM.pdf]

Online-only Methods

Materials and Methods

Chemical compounds and biologic reagents

QA-68-ZU81, EA-89-YM35, and dBRD9-A (VD-83-AX41) were synthesized at Novartis Pharma AG, Basel, Switzerland. 5-azacytidine and decitabine were purchased from Haoyuan chemexpress (Shanghai, China). Ara-c and daunorubicin hydrochloride were purchased from Sigma-Aldrich (St. Louis, MO). ABT-199 (venetoclax; Venclexta), ABT-263 and olaparib (AZD2281) were purchased from Selleckchem (Houston, TX). JQ-1 was purchased from MolPort SIA (Riga, Latvia).

Cell lines and cell culture

MOLM13 and MOLM14 were provided by Dr. Scott Armstrong (Dana-Farber Cancer Institute, MA). MV4,11 and THP-1 cells were obtained from Dr. Anthony Letai (Dana-Farber Cancer Institute, MA). Kasumi-1-luc+ cells were gifts from Dr. Andrew Kung (Memorial Sloan Kettering Cancer Center, NY). HEL, HL60 and K052 cell lines were purchased from ATCC (Manassas, VA). SKM-1 and P31-FUJ cell lines were obtained from Dr. Gary Gilliland (Fred Hutchinson Cancer Research Center, WA).

The human B-ALL lines, SEM, SEMK2, NALM6, RS4;11, and the T-ALL line, PF-382, were provided to us by Dr. David Weinstock (Dana-Farber Cancer Institute, MA). The T-ALL line, JURKAT, was obtained from American Type Culture Collection (ATCC, Manassas, VA). REH, RCH-ACV, 697, and SUP-B15 cells were obtained by Yana Pikman (Dana-Farber Cancer Institute, MA). The human MM lines, H929, MM.1S, U266, and 8226 cell lines were provided to us by Dr. Kenneth Anderson (Dana-Farber Cancer Institute, MA). The T-ALL lines, LOUCY, CCRF-CEM, DND-41,

HPB-ALL, KOPT-K1, and MOLT4, were obtained from Dr. Mark Zimmerman (Dana-Farber Cancer Institute, MA). HCT116 cells were purchased from Horizon Discovery.

AML, MM, and B-ALL cell lines were cultured with 5% CO_2 at 37°C, at a concentration of 2×10^5 to 5×10^5 cells/mL in RPMI 1640 media, purchased from Gibco (Amarillo, TX) (cat #11875-093). Media was supplemented with 10% fetal bovine serum (FBS), purchased from Gibco (Amarillo, TX) (cat # 10437-028) and 1% penicillin/streptomycin (5,000 U/mL), purchased from Gibco (Amarillo, TX) (cat # 15070063). H929 cell media was supplemented with 2-mercaptoethanol (50 μM). The T-ALL lines, LOUCY, CCRF-CEM, DND-41, HPB-ALL, KOPT-K1, and MOLT4 were cultured in RPMI 1640 media, purchased from Gibco (Amarillo, TX) (cat # 11875-093). T-ALL culture media was supplemented with the following, which were purchased from Gibco (Amarillo, TX): 10% FBS (cat # 10437-028), 1% penicillin-streptomycin-glutamine (100X) (cat # 10378-016), 1% NEAA (100X) (cat # 11140-050), 1% 100x sodium pyruvate (100 mM) (cat # 11360-070), 0.1% 1000x 2ME (55 mM) (cat # 21985-023) and 2.5% HEPES (1M) (cat # 15630-080).

Cell lines were submitted for cell line authentication and were authenticated within 6 months of manuscript preparation through cell line short tandem repeat (STR) profiling (Molecular Diagnostics Core, Dana-Farber Cancer Institute). All cell lines tested matched ≥80% with lines listed in the DSMZ Cell Line Bank STR database (https://www.dsmz.de/catalogues/catalogue-human-and-animal-cell-lines/quality-assurance/identity-control/authentication-of-cell-lines.html). All cell lines were confirmed to be virus- and mycoplasma-free.

Chemical compounds and biologic reagents

Ara-c was dissolved in water to obtain a 10 mM stock solution. All other drugs were dissolved in DMSO to obtain a 10 mM stock solution. Serial dilutions were then made, to obtain final dilutions for

cellular assays with a final concentration of DMSO not exceeding 0.1% for those drugs initially dissolved in DMSO.

Normal bone marrow liquid culture proliferation and colony assay studies

Human bone marrow cells obtained from healthy donors were obtained from STEMCELL Technologies INC (Vancouver, British Columbia, Canada). Normal bone marrow was investigated for responsiveness to targeted BRD9 degrader/inhibitor treatment in liquid culture (Iscove's Modified Dulbecco's Medium (MDM) (STEMCELL Technologies INC (Vancouver, British Columbia, Canada) (cat # 36150), supplemented with 20% FBS and StemSpan CC100 serum-free culture supplement for expansion of human hematopoietic cells (100X) (STEMCELL Technologies INC (Vancouver, British Columbia, Canada)).

Normal human bone marrow was also analyzed in a colony assay: 24-well plates seeded with 200 cells/well in MethoCult H4434 Classic (methylcellulose-based medium with recombinant cytokines for human cells) (STEMCELL Technologies INC) (Vancouver, British Columbia, Canada). The plates contained targeted BRD9 degraders/inhibitors at the indicated concentrations. Plates were incubated at 37°C in 5% CO_2 for > 1 week, and then myeloid and erythroid colonies (early progenitors with erythroid and myeloid components: CFU-GM, CFU-E, BFU-E, and CFU-GEMM) were counted on an inverted microscope.

AML patient sample liquid culture proliferation and colony assay studies

Frozen vials of bone marrow from AML patients were previously Ficoll-purified to obtain mononuclear cells and thawed prior to use in studies. AML cells were investigated for responsiveness to targeted BRD9 degrader/inhibitor treatment in liquid culture (Iscove's Modified

Dulbecco's Medium (MDM) (STEMCELL Technologies INC (Vancouver, British Columbia, Canada) (cat # 36150), supplemented with 20% FBS). All bone marrow samples from AML patients were obtained under approval of the Dana-Farber Cancer Institute Institutional Review Board.

Primary AML cells were also analyzed in a colony assay: 24-well plates seeded with 40,000-50,000 cells/well. Cells were placed in 300 uL IMDM media+20% FBS and supplemented with StemSpan™ CC100 serum-free culture supplement for expansion of human hematopoietic cells (100X) (STEMCELL Technologies Inc., Vancouver, British Columbia, Canada) and FLT3L (100 ng/mL) (cat # 130-096-479) (Miltenyi Biotech, Bergisch Gladbach, Germany) and added to 700 uL Enriched MethoCult™ H4435 Enriched Methylcellulose Medium (STEMCELL Technologies, Inc., Vancouver, British Columbia, Canada) or MethoCult H4434 Classic (methylcellulose-based medium with recombinant cytokines for human cells) (STEMCELL Technologies INC) (Vancouver, British Columbia, Canada). Colonies were counted and imaged at the indicated days post-seeding. Colonies were stained as previously described ((1)).

SKM-1 cells (drug-treated as a positive control) were seeded at 100-200 cells/well. Primary AML cells and SKM-1 cells were investigated in parallel in the presence and absence of drug in MethoCult H4434 Classic (STEMCELL Technologies, Inc., Vancouver, British Columbia, Canada). The plates contained targeted BRD9 degraders/inhibitors at the indicated concentrations and were incubated and analyzed (as described above).

Primary MM studies

Effects of BRD9 degrader (BCD9, BCJ1 and BCA1) treatment on proliferation of primary plasma cells from MM patients and normal/healthy subject was performed as service

from Cellatrix LLC, St. Louis, MO. Bone marrow aspirates of 3 MM patients and one healthy donor were used to develop 3D tissue engineered bone marrow (3DTEBM) cultures, as previously described (2). 3DTEBM cultures were treated with increasing concentrations of dBRD9-A (range 0-1000 nM) for five days, then the 3DTEBM cultures were digested, and mononuclear cells were retrieved. Plasma cells were stained with CD138+CD38+ antibodies and analyzed by flow cytometry. MM survival was determined by counting the number of MM cells in each well following treatment, and then normalized to untreated control. (NS= normal subject; ND=newly diagnosed MM; RR= relapsed/refractory MM).

Cell proliferation studies, apoptosis assays, and cell cycle analysis

The Trypan blue exclusion assay was utilized for cell counting prior to seeding for CellTiter-Glo experiments. CellTiter-Glo (Promega, Madison, WI) was used for proliferation studies according to manufacturer's instructions. Cell viability is shown in graphs as the percentage of control (untreated) cells; error bars represent the standard deviation for each data point. Cells infected with GFP tagged short hairpin vectors were followed by GFP and DAPI (F10347, Thermo) on HTS FACS by half passaging at each time point.

Programmed cell death of inhibitor-treated cells was determined using the Annexin-V-Fluos Staining Kit (Boehringer Mannheim, Indianapolis, IN), as previously described (3). Cell cycle analysis was performed as previously described (3).

Drug combination studies

For drug combination assays, we initially quantified cells for seeding using the Trypan Blue exclusion assay. The CellTiter-Glo protocol (per manufacturer's instructions) was then

carried out for proliferation studies. Single agents were added simultaneously at fixed ratios to cells. Cell viability was subsequently expressed as a function of growth affected, drug-treated versus DMSO control cells and data were analyzed by Calcusyn software (Biosoft, Ferguson, MO and Cambridge, UK). This software, which is based on isobologram generation (4), was used for measurement of synergy or antagonism. This approach uses the median effect principle to quantify drug combination effects to assess whether or not they are greater than those expected from a simple addition of the single agent effects. Following estimation of the ED_{50} or IC_{50} of each drug, combinations were analyzed where the concentrations are fractions or multiples of the ED_{50}/IC_{50}. Combination indices or values generated by the Calcusyn software (Biosoft, UK) are either less than one (indicative of synergy) or greater than one (indicative of antagonism).

RNA-seq analysis

Total RNA was isolated from cells using the RNeasy Mini Kit (Qiagen, location?) as per the manufacturer's instructions. Libraries were prepared using Roche Kapa mRNA HyperPrep strand specific sample preparation kits from 200 ng of purified total RNA according to the manufacturer's protocol on a Beckman Coulter Biomek i7. The finished dsDNA libraries were quantified by Qubit fluorometer and Agilent TapeStation 4200. Uniquely dual indexed libraries were pooled in an equimolar ratio and shallowly sequenced on an Illumina MiSeq to further evaluate library quality and pool balance. The final pool was sequenced on an Illumina NovaSeq 6000 targeting 40 million 100-bp read pairs per library at the Dana-Farber Cancer Institute Molecular Biology Core Facilities.

Sequenced reads were aligned to the UCSC hg19 reference genome assembly and gene counts were quantified using STAR (v2.7.3a) (5). Differential gene expression testing was performed by DESeq2 (v1.22.1) (6). RNAseq analysis was performed using the VIPER snakemake pipeline (7).

Differentiation assay

Cells were plated at optimal density in 24-well plates, drug or DMSO (for controls) were added to cells. On days 4, 5, and 6 cells were collected, washed with PBS and stained using standard surface staining protocol for CD11b, CD14 and Zombie aqua. Upregulation of CSF1R and CD36 on cell surface at 72 and 96 hours post drugs treatment was estimated using flow cytometry. All flow cytometry antibodies were purchased from BioLegend (San Diego, CA) unless specified: CD11b (301306), CD14 (367116), CD115 (347312), CD36 (336230) and Zombie Aqua (423102). Data was acquired on BD LSR Fortessa using FACS Diva software and analyzed on Flowjo version 10.6 AND ABOVE (BD biosciences).

Immunoblotting

Protein lysate preparation, immunoblotting, and immunoprecipitation were carried out as has been previously described (3).

Antibodies purchased from Cell Signaling Technologies (Danvers, MA) included anti-GAPDH (14C10) (rabbit mAb, #2118), c-Myb (D2R4Y) (rabbit mAb, #12319), c-Myc (D84C12) (rabbit mAb, #5605), BRD9 (E9R21) (rabbit mAb, #58906). BRD9 ab137245 (rabbit pAb) was purchased from Abcam (Waltham, MA), and BRD9 (rabbit pAb, #61537) was purchased from Active Motif (Carlsbad, CA). The BRD4 antibody (rabbit, Catalog #A301-985A100) was purchased from Bethyl Laboratories (Montgomery, TX). Anti-β-actin antibody

(mouse monoclonal) (clone AC-15, #A1978) was purchased from Millipore-Sigma (Burlington, MA). GAPDH was used at a dilution of 1:3000; all other antibodies were used at a dilution of 1:1000.

The BRD9 polyclonal antibody (24785-1-AP) used for the immunoblot shown in Figure 1C was purchased from Proteintech Group, Inc (Rosemont, IL). This antibody was used at a dilution of 1:1000. Anti-β-tubulin antibody, clone AA2 (05-661) was purchased from Millipore-Sigma (Burlington, MA) and used at a dilution of 1:1000.

Construction of genetic KO of BRD9

The pLentiCRISPRv2 vectors with sgBRD9-1 (5'-CTTGACGGACAGTACCGCAG-3') and with sgBRD9-2 (5'- CTTCGCCAACTTGTAGTACA-3') were obtained from Genescript (Location). The control plasmid pLentiCRISPRv2-sgEGFP which is designed to cut GFP sequence was obtained from Addgene (location). Lentivirus were produced in HEK293T cells using psPAX2 (Addgene #12260) and PMD2.G (Addgene# 12259). Cells were spin infected with freshly collected virus supernatant using polybrene transfection reagent (Sigma # TR1003). Cells were washed six hours post-infection and subjected to puromycin (0.5μg/mL) selection at 24 hours post-infection. Immunoblotting and amplicon sequencing was performed to access knock out efficiency.

Genomic DNA from knockout cells was isolated using DNeasy Blood & Tissue Kit (Qiagen). Forward primer (5'-GTTGATCCTGTCCCGAGCTT-3') and reverse primer (5'-TATGCTCTGCCAGCTCTGTG-3') were used to amplify a 237 bp amplicon around the sgBRD9-1 editing site by PCR. The PCR product was subjected to agarose (1.5%) gel electrophoresis and followed by purification using QIAquick Gel Extraction Kit (Qiagen,

location?). Amplicon sequencing was carried out by Molecular Biology Core Facility at DFCI. The characterization of CRISPR editing was analyzed and visualized using CRISPResso2 (https://crispresso.pinellolab.partners.org/submission).

BRD9 shRNA	Target seqencing	location on NM_023924.4 (CDS:168-1961)	
BRD9-shRNA#1	CGAGTAGAGAAGTTATCAGCT	982-1002	CDS
BRD9-shRNA#2	GAGAGCACACCTATTCAGCAA	576-596	CDS
BRD9-shRNA#4	GCCCTTGTTTCTGCCAGTGTA	2384-2404	3'-UTR
BRD9-shRNA#5	GCAATTGCTCCTGGATATTCA	669-689	CDS

KD studies:

Doxycycline-inducible BRD9 KD: HEK-293T cells were transfected with TransIT-293 Reagent as per manufacturer's protocol. After 24 h cell growth, the medium was replaced and the cells were incubated. Supernatant containing viruses was collected 72 h post-transfection, loaded into a 20 mL syringe and filtered through a 0.45 μm filter. SKM1, cells were transduced by spinfection with the addition of Polybrene at a final concentration of 8 μg/mL. 0.5 μg/mL Puromycin selection involving serial passaging until uninfected control cells succumbed for 2 weeks was performed before subjecting the polyclonal population to fluorescence-activated cell sorting. Briefly, 10^6 cells were counted for each sample, spun down at 500 × g at 4°C for 3 min, washed with 500 μL PBS and resuspended in FACS buffer (PBS supplemented with 4% FBS) and analyzed on M Aria II SORP UV (BD Biosciences, San Jose, USA). Single cell population was selected plotting forward scatter (FCS) versus side scatter (SSC); doublets were excluded from the analysis by plotting FCS width versus FCS area and sorted in a 96-well plate. Selection was applied for 2 weeks to expand the monoclonal population. West Blot analysis confirmed that

doxycycline treatment of 1ug/mL induced knockdown of BRD9 in the clones and not in the sham control.

BRD9 KD: Plko.1 puro vectors containing Shs against BRD9 were purchased from Sigma (location?). Addgene#8453 was used to replace puromycin sequence with GFP using GenScript custom cloning services. Puro was replaced with GFP in these Sh plasmids: TRCN0000128333, TRCN0000127634, TRCN0000127780, TRCN0000131081. High titer lentiviral particles were produced in HEK293T cells, supernatant at 48 and 72 h were pooled and ultra-centrifuged; titers were calculated by percent of GFP positive MV4;11 cells at 48 hours. Cells were spin infected with multiplicity of infection (MOI)5 using polybrene reagent. Knock down was confirmed by western blot and mRNA qPCR.

Chemical synthesis of EA-89-YM35 and QA-68-ZU81

Figure 1a Supplementary

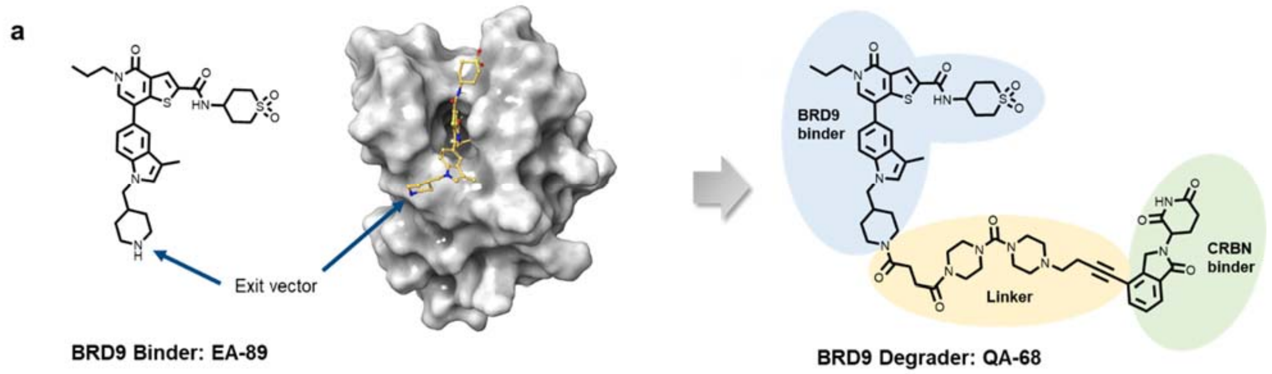

EA-89 represents a structurally novel BRD9 binder that was selected for incorporation into the bifunctional degrader **QA-68** based on an accessible exit vector at the pendant piperidine nitrogen (blue arrow) for linker attachment. The design and optimization of this chemical series through structure-based design will be described in a future chemistry-based publication.

QA-68 could not be progressed for *in vivo* studies owing to very poor high throughput equilibrium solubility (0.0003 g/L at pH 6.8) which limited formulation of the compound to concentrations below 1mg/mL.

Synthesis of EA-89

Scheme-1 (Synthesis of intermediate-1)

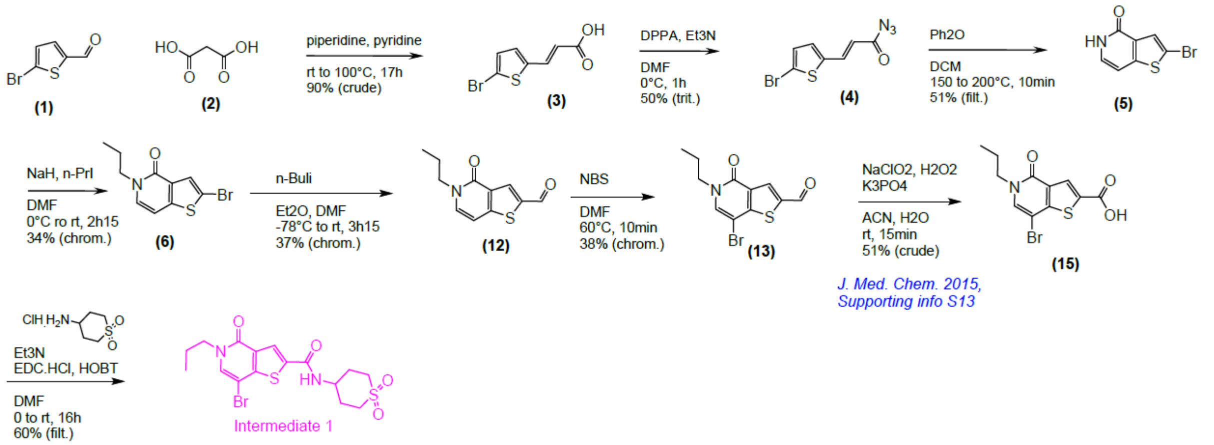

Step-1

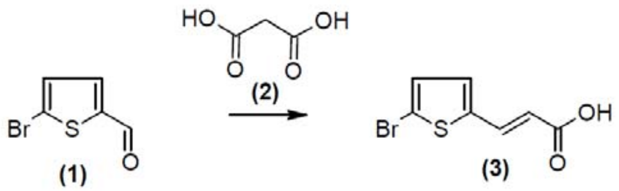

Preparation of compound-3: To a stirred solution of compound- **1** (50.0 g, 261.7 mmol) in piperidine (14 mL) and pyridine (750 mL) was added malonic acid **2** (43.54 g, 418.72 mmol) at RT. After addition, the mixture was heated at 80°C for 1 h and then at 100°C for 16 h. The reaction was monitored by TLC and when the starting material had been consumed, the pyridine was evaporated off and the residue was dissolved in water and acidified with HCl (*pH* = 2). The resulting precipitate was filtered, washed with cold water and dried under vacuum to give the crude acid-**3** (55 g, yield 90%). This was used for the next step without purification.

Step-2

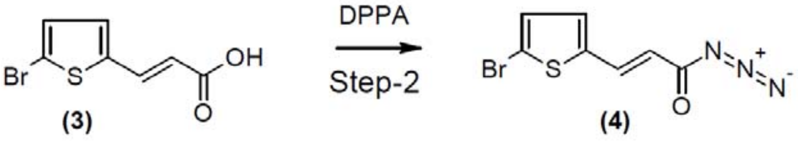

Preparation of compound-4: To a stirred solution of compound-**3** (1.5 g, 6.43 mmol) in DMF (15 mL) at 0°C was added Et_3N (1.07 mL, 7.72 mmol), followed by drop-wise addition of DPPA (1.53 mL, 7.08 mmol) and stirring was continued for 1 h. The reaction was monitored by TLC and when the starting material had been consumed, the mixture was poured into ice-water and the resulting precipitate was filtered and dried carefully under vacuum at low temperature to give the crude compound. The crude compound was then triturated with pentane to give the title compound as a brown solid-**4** (0.83 g, yield 50%), which was used for the next step without further purification.

Step-3

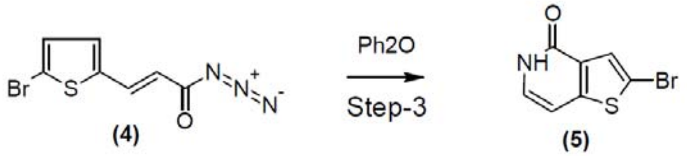

Preparation of compound-5: Compound-**4** (12 g, 46.51 mmol) in DCM (66 mL) was added dropwise to diphenyl ether (60 mL) at 150°C while stirring. The temperature of the mixture was then raised gradually to 200°C and held for10 min. The reaction was monitored by TLC and when the starting material had been consumed, the mixture was cooled and treated with hexane. The resulting precipitate was washed with hexane and ether to give the title compound-**5** (5.4 g, yield 51%), which was used for the next step without further purification.

Step-4

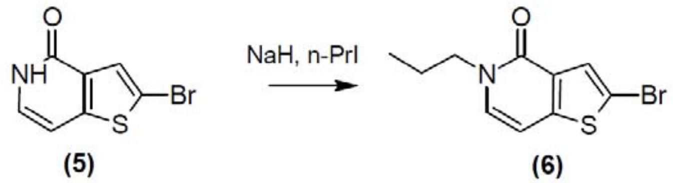

Preparation of compound-6: To a suspension of NaH (4.22 g, 176.08 mmol) in DMF (100 mL) at 0°C was added compound-**5** (27 g, 117.39 mmol) dissolved in DMF (120 mL) under an argon atmosphere. After stirring for 15 min at RT, *n*-PrI (12.61 mL, 129.2 mmol) was added drop-wise and the reaction mixture was stirred for 2 h at RT. The reaction was monitored by TLC and when the starting material had been

consumed, the mixture was poured slowly onto crushed ice and extracted with EtOAc. The organic layer was washed with water, brine and dried over Na_2SO_4. It was then filtered, concentrated and purified over silica gel column using ethyl acetate / hexane as an eluent to give the title compound-**6** (11 g, yield 34%) as a yellow solid.

Step-5

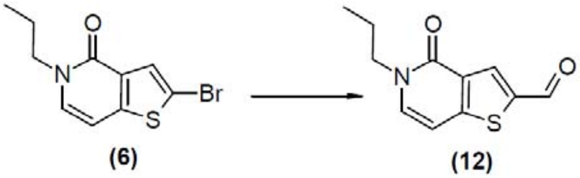

Preparation of compound-12: To a stirred solution of compound-6 (100 mg, 0.36 mmol) in dry ether (3 mL) was added *n*-BuLi (2.2 M) (0.41 mL, 0.91 mmol) drop wise at -78°C and stirring was continued for 15 min at the same temperature. Dry DMF (0.042 mL, 0.55 mmol) was added drop-wise and temperature was allowed to increase to RT and stirring was continued for 3 h. The reaction was monitored by TLC and when the starting material had been consumed, the mixture was quenched by the drop-wise addition of water at 0°C. The ether layer was separated, washed with water, brine and dried over Na_2SO_4. It was then filtered, concentrated and purified by silica gel column chromatography using EtOAc / hexane as an eluent to give the desired compound-**12** (0.03 g, yield 37 %), as a yellow solid.

Step-6

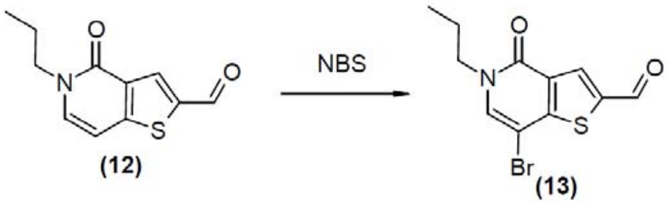

Preparation of compound-13: To a stirred solution of compound-12 (6 g, 27.14 mmol) in dry DMF (60 mL) was added NBS (5.3 g, 29.86 mmol) and the mixture was stirred 10 min at 60°C. The reaction was monitored by TLC and when the starting material had been consumed the mixture was poured into ice-cold water and extracted with ethyl acetate. The combined organic layers were washed with water, brine and dried over Na_2SO_4, the filtered, concentrated and purified by silica-gel column

chromatography using EtOAc / hexane as an eluent to give the desired compound-13 (3.1 g, yield 38%) as a yellow solid.

Step-7

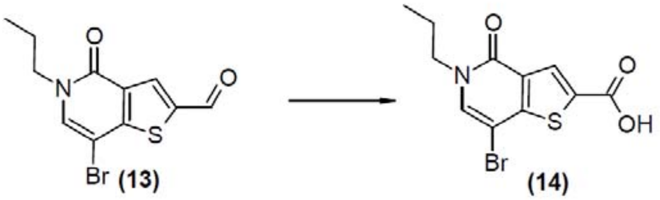

Preparation of compound-14: To a stirred solution of compound-**13** (2.6 g, 8.6 mmol) in acetonitrile (80 mL) was added aqueous hydrogen peroxide (30%; 19.5 mL, 173.3 mmol), a solution of sodium chlorite (4.7 g, 51.82 mmol) and potassium phosphate monobasic (17.6 g, 129.56 mmol) in water (80 mL) at RT. Stirring was then continued for 15 min at RT. Reaction was monitored by TLC and after completion of the reaction, mixture was diluted with EtOAc. The combined organic layers were separated, washed with brine and dried over Na_2SO_4, and then concentrated under reduced pressure to afford the crude compound. The crude compound was then treated with acid and base to give the desired compound-**14** (1.4 g, yield 51%) as a yellow solid.

Step-8

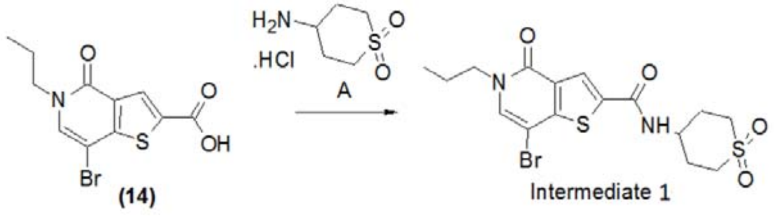

Preparation of Intermediate-1: To a stirred solution of compound-**14** (1.3 g, 4.11 mmol) in dry DMF (10 mL) was added Et_3N (1.42 mL, 10.27 mmol) at 0°C, followed by the addition of EDC.HCl (1.17 mL, 6.16 mmol) and HOBT (0.68 g, 4.56 mmol) at 0°C. The reaction mixture was stirred for 20 min and then reagent-A (0.91 g, 4.93 mmol) was added and stirring was continued for 16 h at RT. The reaction was

monitored by TLC and when the starting material had been consumed, the mixture was poured onto ice-cold water and the precipitate was filtered and dried under reduced pressure to give the desired compound i.e.; Intermediate-1 (1.1 g, yield 60%), as an off-white solid.

Scheme-2 (Synthesis of EA-89)

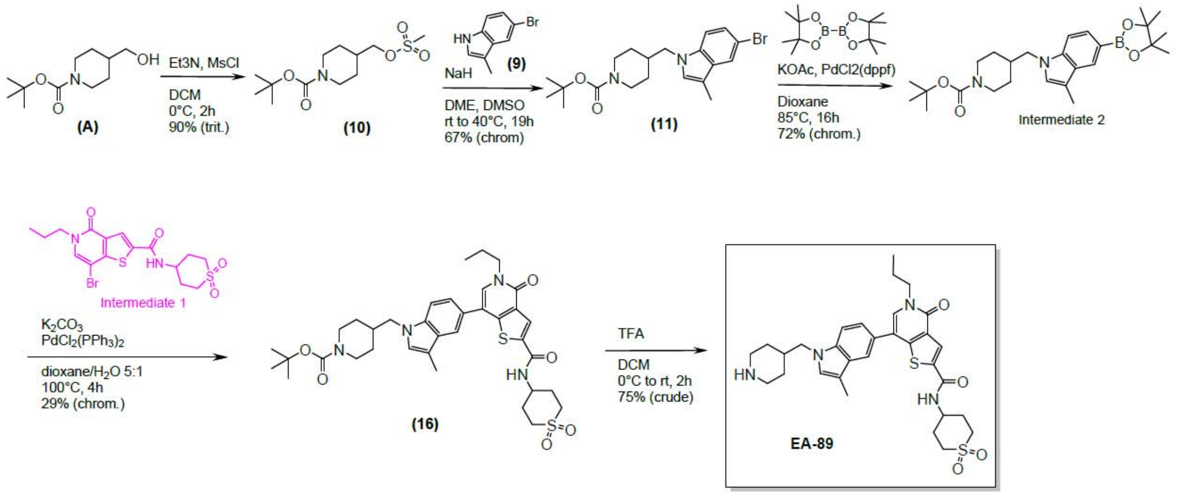

Step-9

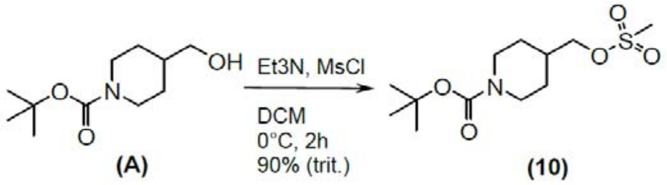

Preparation of compound-10: To a stirred solution of compound-**A** (10 g, 46.5 mmol) in CH_2Cl_2 (100 mL) was added Et_3N (16.2 mL, 116.25 mmol) at 0°C, followed by the drop-wise addition of MsCl (4.3 mL, 55.8 mmol). Stirring was continued for 2 h at 0°C. The reaction was monitored by TLC and when the starting material had been consumed the mixture was diluted with CH_2Cl_2 and washed with water, brine and dried over Na_2SO_4, then filtered and concentrated under reduced pressure to give the crude compound as a gum, which was triturated with pentane to give the crude compound-**10** (12.3 g, yield 90%) as white solid, which was used for the next step without further purification.

Step-10

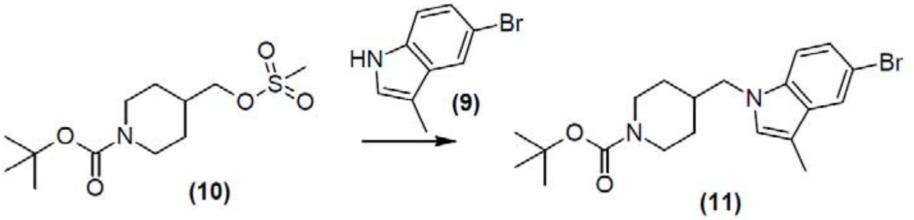

Preparation of compound-11: NaH (60% dispersion in mineral oil; 0.183 g, 4.57 mmol) was added to DME (13 mL) at RT under an argon atmosphere. To this suspension was added DMSO (1.4 mL) followed by compound-9 (0.8 g, 3.81 mmol). Stirring was continued for 30 min at RT and then compound-10 (1.3 g, 4.57 mmol) was added portion-wise. Stirring was continued for 30 min at RT and then the mixture was heated at 40°C for 19 h. The reaction was monitored by TLC and when the starting material had been consumed the, mixture was poured slowly onto crushed ice and then extracted with EtOAc. The combined organic layers were washed with water, brine and dried over Na_2SO_4., and then filtered and concentrated under reduced pressure to give the crude material. The crude material was purified by silica gel column chromatography using EtOAc / hexane as an eluent to give the desired compound-**11** (1.21 g, yield 67%) as a gum.

Step-11

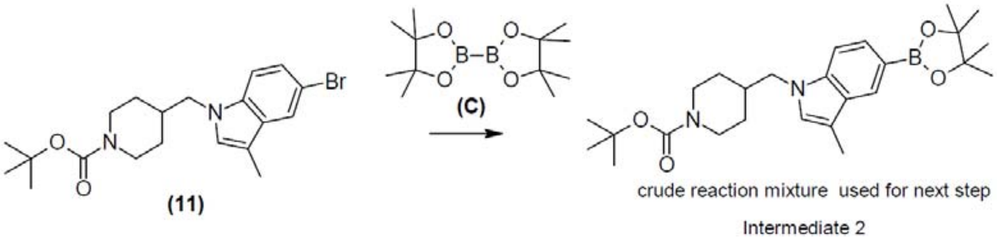

Preparation of Intermediate-2: A mixture of compound-**11** (2.5 g, 6.14 mmol), reagent-C (1.24 g, 4.91 mmol) and KOAc (1.8 g, 18.42) in dioxane (25 mL) was degassed for 20 min under an argon atmosphere. PdCl2(dppf) (314 mg, 0.43 mmol) was then added and degassing was continued for 10 min. The mixture was then heated at 85°C for 16 h. the reaction was monitored by TLC and when the starting material had been consumed the, solvent was evaporated-off under reduced pressure to give the crude material. The crude material was purified by silica-gel column chromatography using EtOAc / hexane as an eluent to give the desired compound i.e.; Intermediate-**2** (2.0 g, yield 72%) as an off-white solid.

Step-12

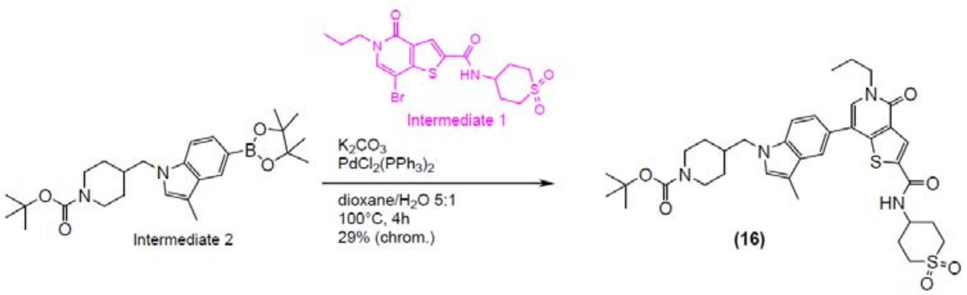

Preparation of compound-16: A mixture of intermediate-**1** (0.5 g, 1.1 mmol), intermediate-2 (0.61 g, 1.34 mmol) and K_2CO_3 (0.45 g, 3.3 mmol), dioxane (7.5 mL) and water (1.5 mL) in a sealed tube was degassed for 15 min under an argon atmosphere. $Pd(PPh_3)_2Cl_2$ (39 mg, 0.05 mmol) was then added and the mixture was heated at 100°C for 4 h. The reaction was monitored by TLC and when the starting material had been consumed the solvent was evaporated off under reduced pressure and the crude product was treated with saturated aqueous $NaHCO_3$ (24 mL) and extracted with EtOAc (36 mL) The combined organic layers were washed with water, brine and dried over Na_2SO_4, filtered and concentrated under reduced pressure to give the crude product which was purified by silica gel column chromatography using acetone / CH_2Cl_2 as an eluent to give the title compound-**16** (0.22 g, yield 29%) as an off-white solid.

Step-13

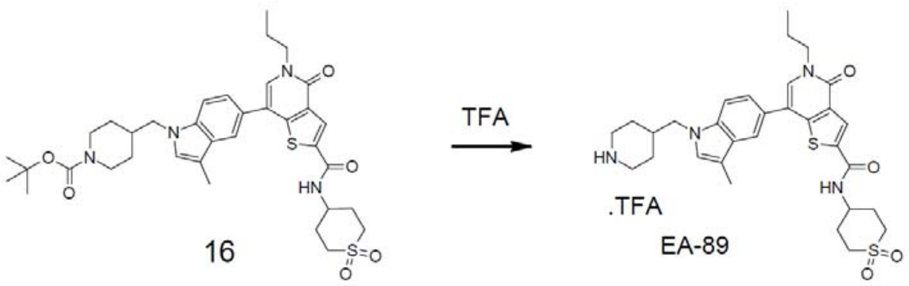

Preparation of EA-89: To a stirred solution of compound- **16** (1.0 g, 1.4 mmol) in CH_2Cl_2 (10 mL) as added TFA (1.1 mL, 14.4 mmol) at 0°C.The mixture was slowly warmed to RT and stirred for 2 h. The reaction was monitored by TLC and when the starting material had been consumed, the solvent was evaporated off to give the crude product, which was treated with water (50 mL) and washed with EtOAc. After aqueous phase was concentrated under reduced pressure to give the desired compound i.e.; **EA-89** (630 mg, yield 75%) as an off-white solid.

Synthesis of QA-68

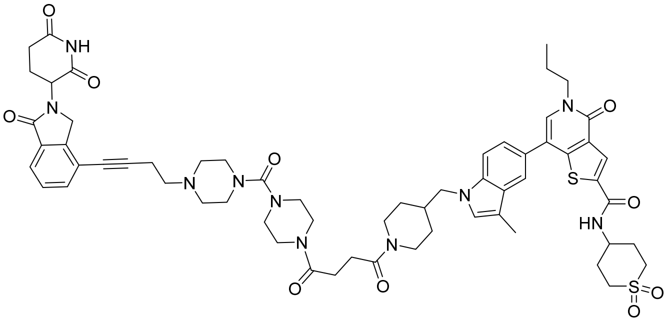

Scheme-3 (Synthesis of intermediate-3)

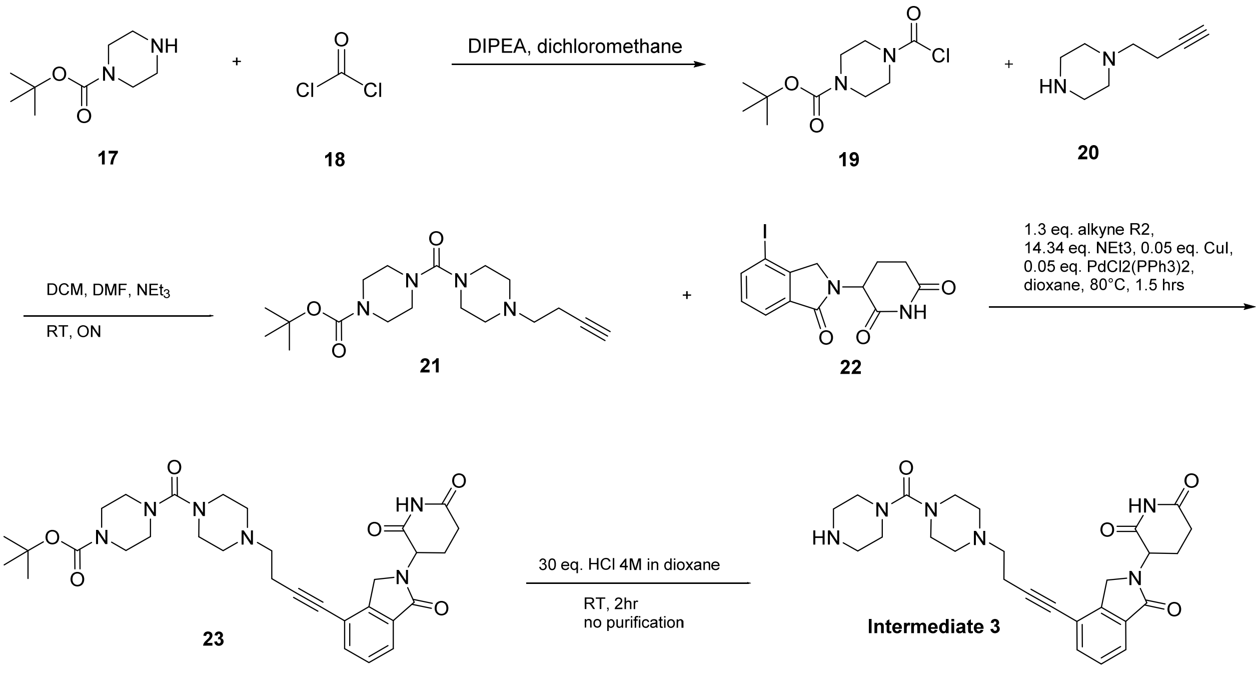

Step 1: Tert-butyl 4-(chlorocarbonyl)piperazine-1-carboxylate

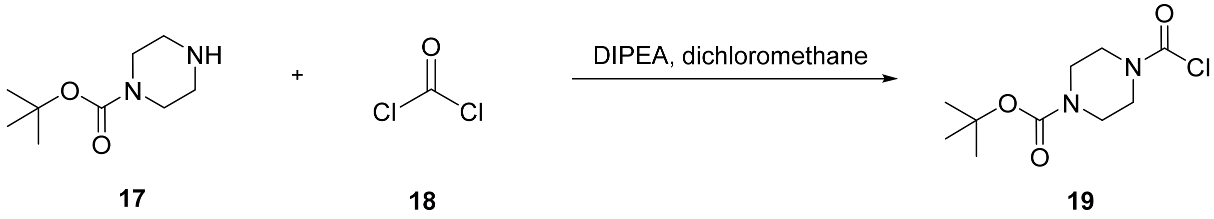

Preparation of compound-19: The reaction vessel was charged with 400 mL DCM and the phosgene solution **18** (85 g, 859 mmol). The mixture was cooled to 0°C and a solution from Boc piperazine **17** (80 g, 430 mmol) and DIPEA (61.1 g, 472 mmol) in CH_2Cl_2 (500 mL) was added dropwise in 50 minutes. The

mixture was stirred for 1 h at 0°C, then DIPEA (75 mL) was added dropwise over 5 min and was followed by stirring for 2 h at 0°C to 10°C. The reaction was evaporated to dryness giving 147g of crude product as a beige solid. Caution: Phosgene, use gas trap with NaOH. The crude product was purified by silica column eluting with dichloromethane/Ethyl acetate (0 to 2%) giving product **19** (82g, 68% yield)

Step 2: Tert-butyl 4-(4-(but-3-yn-1-yl)piperazine-1-carbonyl)piperazine-1-carboxylate

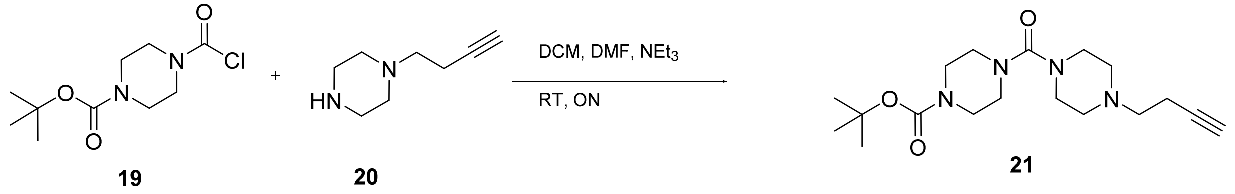

Preparation of compound-21: 1-(but-3-in-1-yl)piperazine **20** (6.0 g, 28.4 mmol) was suspended in the DMF and cooled to 0°C and this was followed by addition of NEt_3 (11.62 g, 115 mmol). tert-butyl 4-(chlorocarbonyl)piperazine-1-carboxylate **19** (7.0 g, 28.1 mmol) dissolved in the DCM was added dropwise to the suspension over 15 min and the reaction was kept below 12°C. The reaction was stirred at RT overnight and diluted with 500 mL DCM, extracted with 500 mL water (x3). The combined water phases were re-extracted twice with 200 mL DCM. The combined organics were dried over $MgSO_4$ and evaporated to dryness giving **21** as a beige-brown solid (82 g, 80% yield) LCMS (m/z): 350.5 $[M + H]^+$.

Step 3: Tert-butyl 4-(4-(4-(2-(2,6-dioxopiperidin-3-yl)-1-oxoisoindolin-4-yl)but-3-yn-1-yl)piperazine-1-carbonyl)piperazine-1-carboxylate

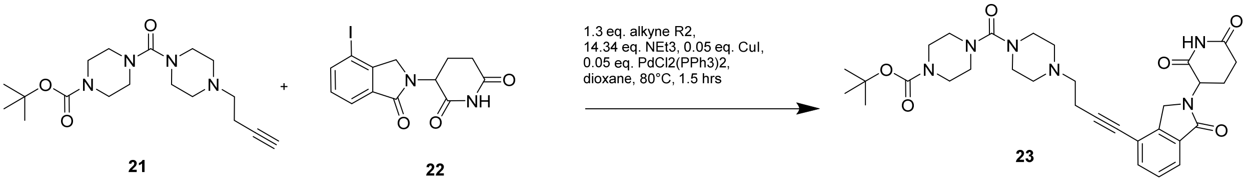

Preparation of compound-23: 3-(4-iodo-1-oxoisoindolin-2-yl)piperidine-2,6-dione **22** (30 g, 81 mmol) was suspended in Dioxane (Volume: 270 mL) under argon. To resulting white slurry was added tert-butyl 4-(4-(but-3-yn-1-yl)piperazine-1-carbonyl)piperazine-1-carboxylate **21** (41.0 g, 105 mmol) with vigorous stirring. Next CuI (0.772 g, 4.05 mmol) and $Pd(PPh_3)2Cl_2$ (2.84 g, 4.05 mmol) were added followed by dropwise addition of NEt_3 (162 mL, 1162 mmol) (250mL-dropping funnel) over 10 min. The thick yellow-brown suspension was vigorously stirred at 85°C for 2 h. The resulting brown suspension was diluted with 1500mL DCM and extracted once with 1000 mL water and once with 1000mL brine. All water

phases were re-extracted once with 1000mL DCM. All DCM phases were combined and dried over $MgSO_4$ and evaporated to dryness. The crude product was purified by silica chromatography eluting with dichloromethane/Ethyl acetate (0 to 20%) giving **23** as an off-white solid (43.5 g, 89% yield). LCMS (m/z): 593.3 $[M + H]^+$.

Step 4: 3-(1-oxo-4-(4-(4-(piperazine-1-carbonyl)piperazin-1-yl)but-1-yn-1-yl)isoindolin-2-yl)piperidine-2,6-dione

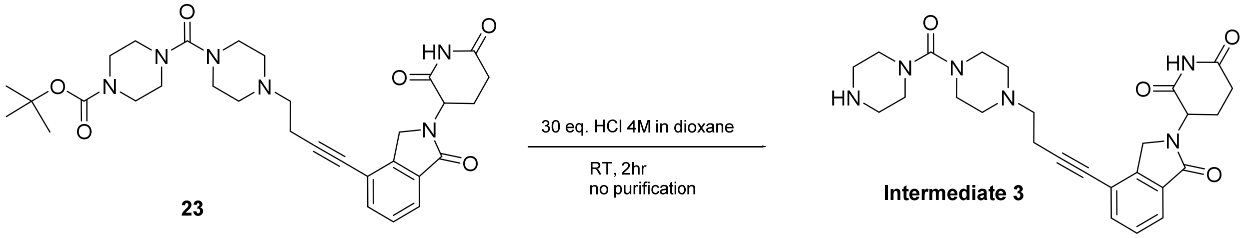

Preparation of Intermediate 3: tert-butyl 4-(4-(4-(2-(2,6-dioxopiperidin-3-yl)-1-oxoisoindolin-4-yl)but-3-yn-1-yl)piperazine-1-carbonyl)piperazine-1-carboxylate **23** (43 g, 72.6 mmol) and HCl in Dioxan (550 ml, 2198 mmol) were mixed and the resulting suspension was stirred under argon at RT for 5.5h. The reaction mixture was diluted with 1600 mL TBME and stirred for 15 minutes at RT. The suspension was filtered off and the filtercake was washed twice with 250 ml TBME, then dried at 30°C in a vacuum drying cabinet (overnight) giving **Intermediate 3** as a beige solid (40.8 g, 98% yield). LCMS (m/z): 493.4 $[M + H]^+$.

Scheme-4 (Synthesis of QA-68)

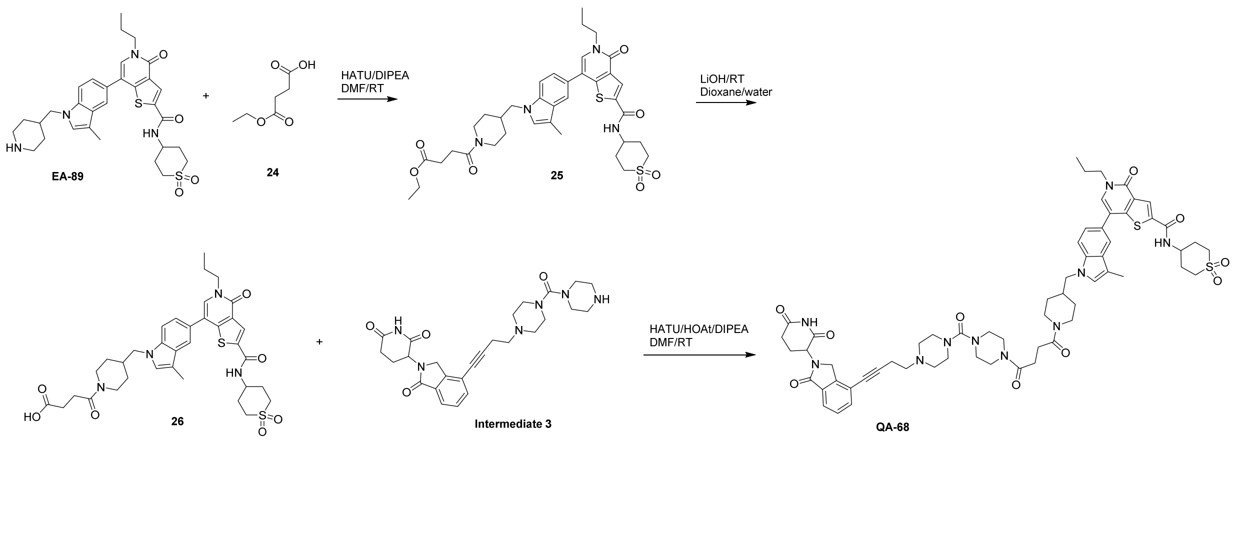

Step 5: Ethyl 4-(4-((5-(2-((1,1-dioxidotetrahydro-2H-thiopyran-4-yl)carbamoyl)-4-oxo-5-propyl-4,5-dihydrothieno[3,2-c]pyridin-7-yl)-3-methyl-1H-indol-1-yl)methyl)piperidin-1-yl)-4-oxobutanoate

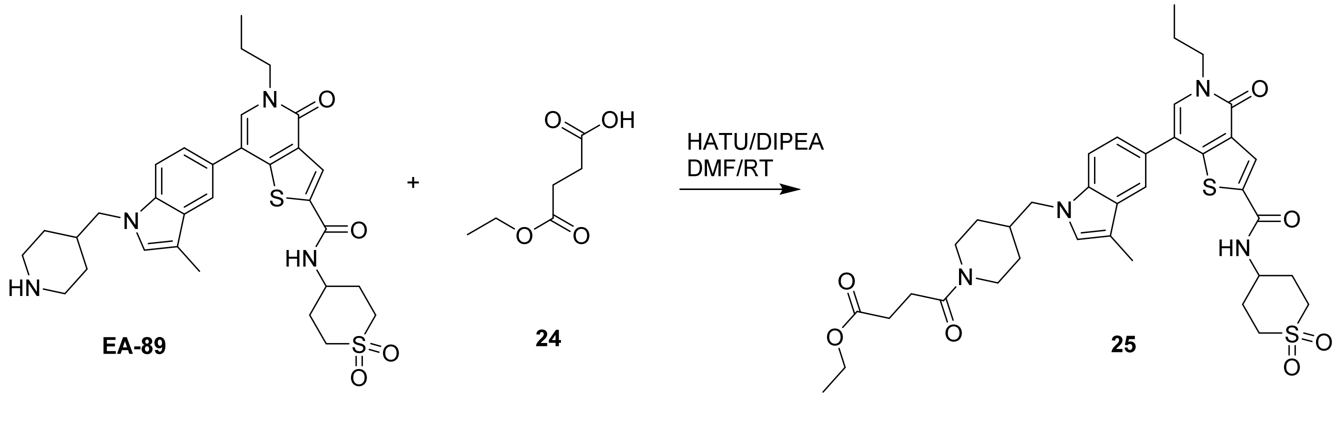

Preparation of compound-25: N-(1,1-dioxidotetrahydro-2H-thiopyran-4-yl)-7-(3-methyl-1-(piperidin-4-ylmethyl)-1H-indol-5-yl)-4-oxo-5-propyl-4,5-dihydrothieno[3,2-c]pyridine-2-carboxamide **EA-89** (0.074 mmol, 52.5 mg) and mono-ethyl succinate **24** (0.084 mmol, 12.3 mg) were dissolved in 0.5 mL of DMF under Ar and followed by addition of HATU (0.148 mmol, 56.3 mg), DIPEA (0.4 mmol, 51.8 mg). The reaction mixture was stirred at RT and after 3h15min, LC-MS shows loss of **EA-89** and conversion to a single new peak with the expected mass. The reaction was worked up diluting with water and extracting with EtOAc. The organic phase was washed with brine 2 times, dried over Na_2SO_4, filtered and evaporated. It was then dried under vacuum overnight to afford **25** as a film (64 mg, 100% yield). LCMS (m/z): 723.5 $[M + H]^+$. The crude product was used for the next step without purification.

Step 6: 4-(4-((5-(2-((1,1-dioxidotetrahydro-2H-thiopyran-4-yl)carbamoyl)-4-oxo-5-propyl-4,5-dihydrothieno[3,2-c]pyridin-7-yl)-3-methyl-1H-indol-1-yl)methyl)piperidin-1-yl)-4-oxobutanoic acid

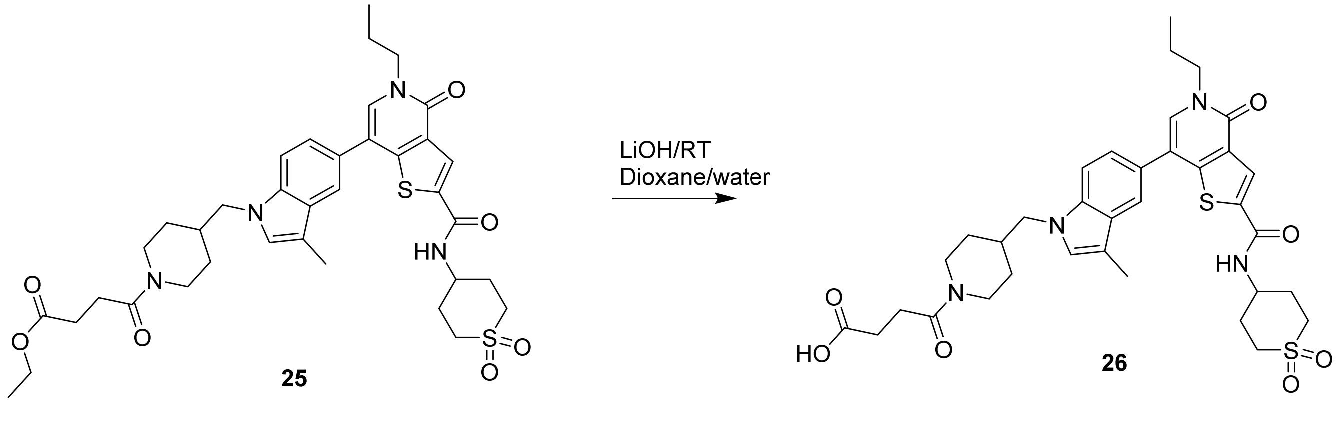

Preparation of compound-26: Ethyl 4-(4-((5-(2-((1,1-dioxidotetrahydro-2H-thiopyran-4-yl)carbamoyl)-4-oxo-5-propyl-4,5-dihydrothieno[3,2-c]pyridin-7-yl)-3-methyl-1H-indol-1-yl)methyl)piperidin-1-yl)-4-oxobutanoate **25** (0.081 mmol, 58.9 mg) and lithium hydroxide (0.122 mmol, 5.1 mg) were dissolved in 0.5 mL of dioxane and 1 mL of water. The reaction was stirred at RT and after 2h LC-MS showed the expected product and no more **25**. The mixture was acidified with HCl 2M, evaporated to dryness and was then dried under vacuum overnight to afford **26** as a yellow residue (66 mg, 94% yield)
LCMS (m/z): 695.4 $[M + H]^+$.

Step 7: N-(1,1-dioxidotetrahydro-2H-thiopyran-4-yl)-7-(1-((1-(4-(4-(4-(4-(2-(2,6-dioxopiperidin-3-yl)-1-oxoisoindolin-4-yl)but-3-yn-1-yl)piperazine-1-carbonyl)piperazin-1-yl)-4-oxobutanoyl)piperidin-4-yl)methyl)-3-methyl-1H-indol-5-yl)-4-oxo-5-propyl-4,5-dihydrothieno[3,2-c]pyridine-2-carboxamide

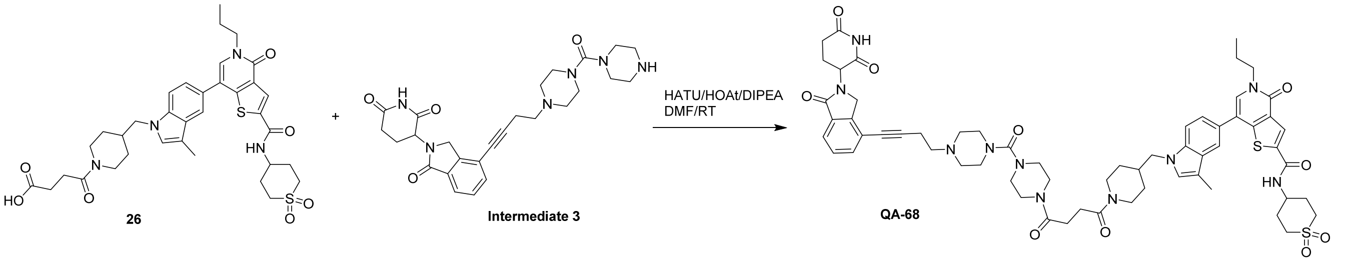

Preparation of QA-68: HATU (0.035 mmol, 13.3 mg), HOAt (0.035 mmol, 4.76 mg)and 4-(4-((5-(2-((1,1-dioxidotetrahydro-2H-thiopyran-4-yl)carbamoyl)-4-oxo-5-propyl-4,5-dihydrothieno[3,2-c]pyridin-7-yl)-3-methyl-1H-indol-1-yl)methyl)piperidin-1-yl)-4-oxobutanoic acid **26** (0.035 mmol, 24.3 mg) were dissolved in 0.5 mL of DMF. This mixture was stirred at RT for 5 min followed by addition of 3-(1-oxo-4-(4-(4-(piperazine-1-carbonyl)piperazin-1-yl)but-1-yn-1-yl)isoindolin-2-yl)piperidine-2,6-dione **Intermediate 3** (0.035 mmol, 19.8 mg) and DIPEA (0.229 mmol, 29.6 mg). The reaction was stirred at RT for 1.25 h and LC-MS showed complete loss of **26** and one product corresponding the expected mass. The mixture was diluted with aq. NH_4Cl and extracted with EtOAc. The organics were evaporated to dryness, dissolved in DMF and purified by preparative HPLC (5-95% AcCN in water in 20 min, with 0.1% of TFA). AcCN was removed from the collected fractions under vacuum and the solution was then freeze-dried overnight to afford a white residue **QA-68** as a TFA salt. (18 mg, 38.5% yield). LCMS (m/z): 1170.7 $[M + H]^+$.

References

1. Borowicz S, Van Scoyk M, Avasarala S, Karuppusamy Rathinam MK, Tauler J, Bikkavilli RK, et al. The soft agar colony formation assay. J Vis Exp. 2014(92):e51998.
2. de la Puente P, Muz B, Gilson RC, Azab F, Luderer M, King J, et al. 3D tissue-engineered bone marrow as a novel model to study pathophysiology and drug resistance in multiple myeloma. Biomaterials. 2015;73:70-84.
3. Weisberg E, Boulton C, Kelly LM, Manley P, Fabbro D, Meyer T, et al. Inhibition of mutant FLT3 receptors in leukemia cells by the small molecule tyrosine kinase inhibitor PKC412. Cancer Cell. 2002;1(5):433-43.
4. Chou TC, Talalay P. Quantitative analysis of dose-effect relationships: the combined effects of multiple drugs or enzyme inhibitors. Adv Enzyme Regul. 1984;22:27-55.
5. Dobin A, Davis CA, Schlesinger F, Drenkow J, Zaleski C, Jha S, et al. STAR: ultrafast universal RNA-seq aligner. Bioinformatics. 2013;29(1):15-21.
6. Love MI, Huber W, Anders S. Moderated estimation of fold change and dispersion for RNA-seq data with DESeq2. Genome Biol. 2014;15(12):550.
7. Cornwell M, Vangala M, Taing L, Herbert Z, Koster J, Li B, et al. VIPER: Visualization Pipeline for RNA-seq, a Snakemake workflow for efficient and complete RNA-seq analysis. BMC Bioinformatics. 2018;19(1):135.

BRD9 complex with EA-89

Protein Purification

The BRD9 construct (amino acid 130-240) was expressed in E.coli BL21(DE3) RIL. The resulting protein was purified by metal chelation and size exclusion chromatography and concentrated by ultrafiltration to 12.4 mg/mL in 25 mM Hepes pH 7.5, 300mM NaCl, 0.5mM TCEP.

Crystallization

Purified protein buffered in 25mM Hepes pH 7.5, 300mM NaCl, 0.5mM TCEP at a concentration of 12.4mg/ml containing 2mM EA-89 was crystallized. 0.2µl protein solution was mixed with 0.2µl well solution and equilibrated against 80µl reservoir using SWISSCI MRC 2 Well crystallization plates designed in the 96 well plate format and sealed with Hampton Research ClearSeal Film. Crystallization plates were incubated at 4°C. Crystals were found under several conditions after 1 week, while crystals suitable for X-ray diffraction experiments were obtained after 3 weeks in 15% PEG 3350, 0.1M Succinic Acid. Crystals were vitrified by plunging them directly into liquid nitrogen.

Data collection and structure determination

Data sets were collected at 1.0Å wavelength with a PILATUS 6M detector at the Swiss Light Source beamline X10SA (Villigen, Switzerland). Data were collected by Expose GmbH. Diffraction images were processed and scaled using XDS and XSCALE, respectively. Structures were solved by molecular replacement (Phaser). The initial model was subjected to iterative cycles of manual rebuilding and subsequent structure refinement in Coot and autoBuster, respectively. The ligand structure was built into unbiased Fo-Fc difference electron density calculated by autoBuster. Final structure refinement statistics are summarized in Supplementary Table 2. Refined coordinates were deposited to the PDB with entry number 8A7I.

Literature:

Kabsch, W. XDS Acta Crystallogr., Sect. D: Biol. Crystallogr. 2010, D66, 125– 132

McCoy, A. J.; Grosse-Kunstleve, R. W.; Adams, P. D.; Winn, M. D.; Storoni, L. C.; Read, R. J. Phaser crystallographic software J. Appl. Crystallogr. 2007,40, 658– 674

Emsley, P.; Lohkamp, B.; Scott, W. G.; Cowtan, K. Features and development of Coot Acta Crystallogr., Sect. D: Biol. Crystallogr. 2010, D66, 486–501

Vonrhein, C., Flensburg, C., Keller, P., Sharff, A., Smart, O., Paciorek, W., Womack, T. & Bricogne, G. (2011). Data processing and analysis with the autoPROC toolbox. Acta Cryst. D67, 293-302

The PyMOL Molecular Graphics System, Version 1.5.0.4; Schrödinger, LLC, 2012.

Wavelength (Å)	1
Resolution range (Å)	34.71-1.76 (1.823 - 1.76)
Space group	P 1 21 1
a, b, c (Å)	36.1 66.4 55.9
α, β, γ (°)	90 105.8 90
Total reflections	40540 (4664)

Unique reflections	21364 (2507)
R-merge	0.020 (0.236)
R-meas	0.028 (0.334)
I / σ(I)	17.55 (2.57)
Completeness (%)	84.54 (99.64)
Multiplicity	1.9 (1.9)
Wilson B-factor (Å^2)	26.1
CC1/2	1 (0.914)
CC*	1 (0.977)
Reflections used in refinement	21347 (2501)
Reflections used for R-free	1013 (122)
R-work	0.193 (0.3009)
R-free	0.227 (0.3257)
CC(work)	0.958 (0.901)
CC(free)	0.940 (0.883)
Number of non-hydrogen atoms	1851
macromolecules	1581
ligands	82
solvent	188
Protein residues	202
R.m.s. deviation bond length (Å)	0.014
R.m.s. deviation angles (°)	1.40
Ramachandran statistics	
Favored (%)	100.00
Allowed (%)	0.00
Outliers (%)	0.00
Rotamer outliers (%)	1.86
Clashscore	0.31
Average B-factor (Å^2)	33.13
Macromolecules (Å^2)	32.14
Ligands (Å^2)	33.35
Solvent (Å^2)	41.31

Supplementary Table 2: Data collection and refinement statistics of BRD9-EA89 complex structure. Numbers in brackets correspond to the high-resolution shell.
